# Use of species’ responses to cryptic anthropogenic disturbances for monitoring biodiversity outcomes in tropical forests

**DOI:** 10.1111/cobi.70159

**Published:** 2025-10-10

**Authors:** Lucy Perera‐Romero, Roan McNab, Rony Garcia‐Anleu, John Polisar, Chris Sutherland, Daniel Thornton

**Affiliations:** ^1^ School of the Environment Washington State University Pullman Washington USA; ^2^ Guatemala Program Wildlife Conservation Society Petén Guatemala; ^3^ Department of Environment and Development, Zamorano Biodiversity Center Zamorano University Tegucigalpa Honduras; ^4^ Wildlife Conservation Society Bronx New York USA; ^5^ Sierra National Forest Clovis California USA; ^6^ Centre for Research into Ecological and Environmental Modelling University of St Andrews St Andrews UK

**Keywords:** biodiversity outcomes, camera trapping, community‐managed forest, cryptic disturbances, forest stewardship council, multispecies occupancy models, protected areas, áreas protegidas, bosque con gestión comunitaria, cámaras trampa, concejo de administración forestal, modelos de ocupación multiespecie, perturbaciones ocultas, resultados de biodiversidad, 隐性干扰, 多物种占域模型, 红外相机监测, 生物多样性结果, 森林管理委员会, 社区管理的森林, 保护地

## Abstract

Measuring area‐based conservation outcomes in tropical forests is challenging due to cryptic human disturbances (e.g., hunting). As a result, comparative studies of management strategies providing quantitative outcomes remain scarce, especially in the Neotropics. We compared species distribution and richness of terrestrial wildlife in a community‐managed forest and a strictly protected area in Guatemala's Maya Biosphere Reserve. Using multispecies occupancy models and a spatially extensive camera‐trapping grid, we assessed how species respond to structural habitat variables (e.g., elevation, forest canopy height), protection, and human access, a proxy for hunting and cryptic resource use (i.e., human activities that occur under the forest canopy) by nearby communities. During 2018–2019, we recorded 26 terrestrial vertebrate species of >1 kg. We found no differences in species richness or mean community occupancy (i.e., average occupancy probability of all species in the community) at the community level between the community‐managed forest and the protected area. For some species, the effects of human access on occupancy were larger than the combined effects of all other habitat variables. In the community‐managed forest, ease of human access negatively influenced the occupancy of tapirs (*Tapirus bairdii*) and hunted species, such as the great curassow (*Crax rubra*), ocellated turkey (*Meleagris ocellata*), and white‐lipped peccary (*Tayassu pecari*). Small and more generalist species were positively affected by ease of access, possibly reflecting trophic release near human settlements. Although large carnivore occupancy was not affected by access in the community‐managed forest, low detection probabilities could reflect density or behavioral changes. These findings illustrate the influence of cryptic disturbances on some species’ distribution in intact forests and suggest that management actions in the community‐managed forest may have helped maintain diverse assemblages. Our study suggests the need to go beyond remotely sensed measures and species richness metrics when assessing and monitoring biodiversity outcomes in tropical forests.

## Abstract (es)

Uso de las respuestas de las especies ante las perturbaciones antropogénicas ocultas para el monitoreo de los resultados de biodiversidad en los bosques tropicales

**Resumen:**

La medición de los resultados de la conservación basada en el área en los bosques tropicales es un reto debido a las perturbaciones humanas ocultas (por ejemplo, la caza). Como resultado, los estudios comparativos de estrategias de gestión que proporcionan resultados cuantitativos siguen siendo escasos, especialmente en el Neotrópico. Comparamos la distribución y la riqueza de especies de fauna terrestre en un bosque con gestión comunitaria y en un área estrictamente protegida en la Reserva de la Biosfera Maya de Guatemala. Utilizamos modelos de ocupación multiespecie y una red de cámaras trampa de gran extensión espacial para evaluar cómo responden las especies a las variables estructurales del hábitat (por ejemplo, la altitud y la altura del dosel forestal), la protección y el acceso humano, un indicador de la caza y el uso críptico de los recursos (es decir, las actividades humanas que se realizan bajo el dosel forestal) por parte de las comunidades cercanas. Entre 2018 y 2019, registramos 26 especies de vertebrados terrestres de más de 1kg. No encontramos diferencias en la riqueza de especies ni en la ocupación media de la comunidad (es decir, la probabilidad media de ocupación de todas las especies de la comunidad) a nivel comunitario entre el bosque gestionado por la comunidad y el área protegida. En algunas especies, los efectos del acceso humano sobre la ocupación fueron mayores que los efectos combinados de todas las demás variables del hábitat. En el bosque gestionado por la comunidad, la facilidad de acceso humano influyó negativamente en la ocupación de tapires (*Tapirus bairdii*) y especies cazadas, como el paují grande (*Crax rubra*), el pavo ocelado (*Meleagris ocellata*) y el pecarí de labios blancos (*Tayassu pecari*). Las especies pequeñas y más generalistas se vieron afectadas positivamente por la facilidad de acceso, lo que posiblemente refleje una liberación trófica cerca de los asentamientos humanos. Aunque la ocupación de los grandes carnívoros no se vio afectada por el acceso en el bosque gestionado por la comunidad, las bajas probabilidades de detección podrían reflejar cambios en la densidad o el comportamiento. Estos hallazgos ilustran la influencia de las perturbaciones ocultas en la distribución de algunas especies en bosques intactos y sugieren que las medidas de gestión en el bosque gestionado por la comunidad pueden haber contribuido a mantener la diversidad de los conjuntos. Nuestro estudio sugiere la necesidad de ir más allá de las medidas de teledetección y los parámetros de riqueza de especies al evaluar y supervisar los resultados de la biodiversidad en los bosques tropicales.

## 利用物种对隐性人为干扰的响应监测热带森林生物多样性保护成效

**【摘要】** 受到隐性人为干扰(如狩猎)的影响, 监测热带森林中基于区域的保护成效具有挑战性。因此, 提供定量结果的管理策略比较研究仍较为少见, 尤其是在新热带地区。本研究比较了危地马拉玛雅生物圈保护区内由社区管理的森林和严格保护区内陆生野生动物的物种分布情况和丰富度。我们利用多物种占域模型和广泛布设的红外相机网格, 评估了物种对结构性栖息地变量(如海拔、林冠高度)、保护, 以及人类接近程度(代表附近社区的狩猎和隐性资源使用, 即发生在林冠下的人类活动)的响应。在2018‐2019年间, 我们记录到了26种体重大于1千克的陆生脊椎动物。我们发现, 在社区管理的森林和保护区之间, 群落水平上的物种丰富度和平均群落占域率(即群落中所有物种的平均占域率)没有明显差异。对于某些物种而言, 人类的接近对占域率的影响大于所有其他栖息地变量的总体影响。在社区管理的森林中, 人类可接近性对貘(*Tapirus bairdii*)和被狩猎物种(如大凤冠雉[*Crax rubra*]、眼斑吐绶鸡[*Meleagris ocellata*]和白唇西猯[*Tayassu pecari*])的占域率存在负面影响。小型和更生态泛化的物种受到人类可接近性的积极影响, 这可能反映了人类居住区附近的营养级释放。虽然在社区管理的森林中, 大型食肉动物的占域情况未收到人类可接近性的影响, 但这些动物的低监测率可能反映了其密度或行为的变化。这些发现说明了隐性干扰对一些物种在原生林中分布的影响, 并表明社区管理森林中的管理措施可能有助于维持群落多样性。我们的研究表明, 在评估和监测热带森林生物多样性保护成效时, 需要使用遥感测量和物种丰富度指标以外的其他方法。**【翻译:胡怡思;审校:聂永刚】**

## INTRODUCTION

Measuring the effectiveness of conservation strategies relative to impact indicators requires comparing the current state of biodiversity targets with a counterfactual scenario (Pressey et al., 2021). An alternative to a counterfactual approach is to assess outcome‐related indicators, that is, how conservation strategies maintain or enhance the state of biodiversity targets over time. In practice, outcome‐related indicators are used to set goals, are monitored, and are relayed in progress reports (Visconti et al., 2019). Accurate estimates of such metrics are the first step to assessing impact‐related effectiveness of conservation (Pressey et al., 2021). These estimates are critical for local management and global efforts to guide information‐driven fund allocation, actions, international agreements, and national conservation policies.

In the case of tropical forests with intact canopies, the ability of protection and management (Adams et al., 2023; Arneth et al., 2023; Di Girolami et al., 2023) to deliver positive biodiversity outcomes depends on understanding how species and communities respond to cryptic disturbances (Peres et al., 2006). Cryptic disturbances are human activities that do not disturb habitat structure (e.g., forest canopies) and thus are difficult or impossible to assess via remote sensing methods. These activities include hunting, selective logging, extraction of nontimber forest products, and tourism, among others (Larson et al., 2016). Although they occur under relatively intact canopies, cryptic disturbances can significantly affect wildlife communities (Barlow et al., 2016; Benítez‐Lopez et al., 2019; Rija et al., 2020). Robust quantitative assessments of the effects of these disturbances are few due to limited access and the high cost of extensive surveys and the difficulty in detecting species, mainly medium and large terrestrial vertebrates.

Assessing the impact of cryptic disturbances on species is especially pertinent for forest management strategies that purport to have positive biodiversity benefits. Forest Stewardship Council (FSC) reduced‐impact logging certification offers economic incentives to rural communities (Burivalova et al., 2019) and delivers conservation benefits not associated with uncertified forests (Zwerts et al., 2024). However, evidence for the impact of certified forests on medium and large terrestrial vertebrates, based on approaches that include a comparator and account for detection probability and other sources of variability, remains sparse and limited to African and Asia wildlife communities (Matias et al., 2024). Hunting in certified forests is managed at the national level, which means biodiversity outcomes related to hunting depend on national policies. Subsistence hunting occurs in some certified forests, which could affect conservation goals because hunting is often the most critical factor in the status of terrestrial bird and mammal species (Keane et al., 2005; Ripple et al., 2016).

We sought to provide robust measures of biodiversity outcomes in Guatemala's Maya Biosphere Reserve, which is subject to various conservation strategies, including reduced impact logging and strictly protected areas, and has gradients in the intensity of anthropogenic forest use. We used hierarchical, multispecies occupancy models derived from spatially extensive camera‐trapping surveys to assess how cryptic human disturbances affect the distribution and richness of terrestrial species. Our study areas were 2 largely intact forests: an FSC‐certified community‐managed forest subject to subsistence hunting and a formally protected area (reference site). We used human access as a proxy for potential cryptic disturbances, primarily hunting and resource utilization, by people from nearby communities. Species distribution in the community‐managed forest and protected area is likely to differ due to human impacts that vary in intensity based on the distance and size of nearby communities.

**FIGURE 1:**
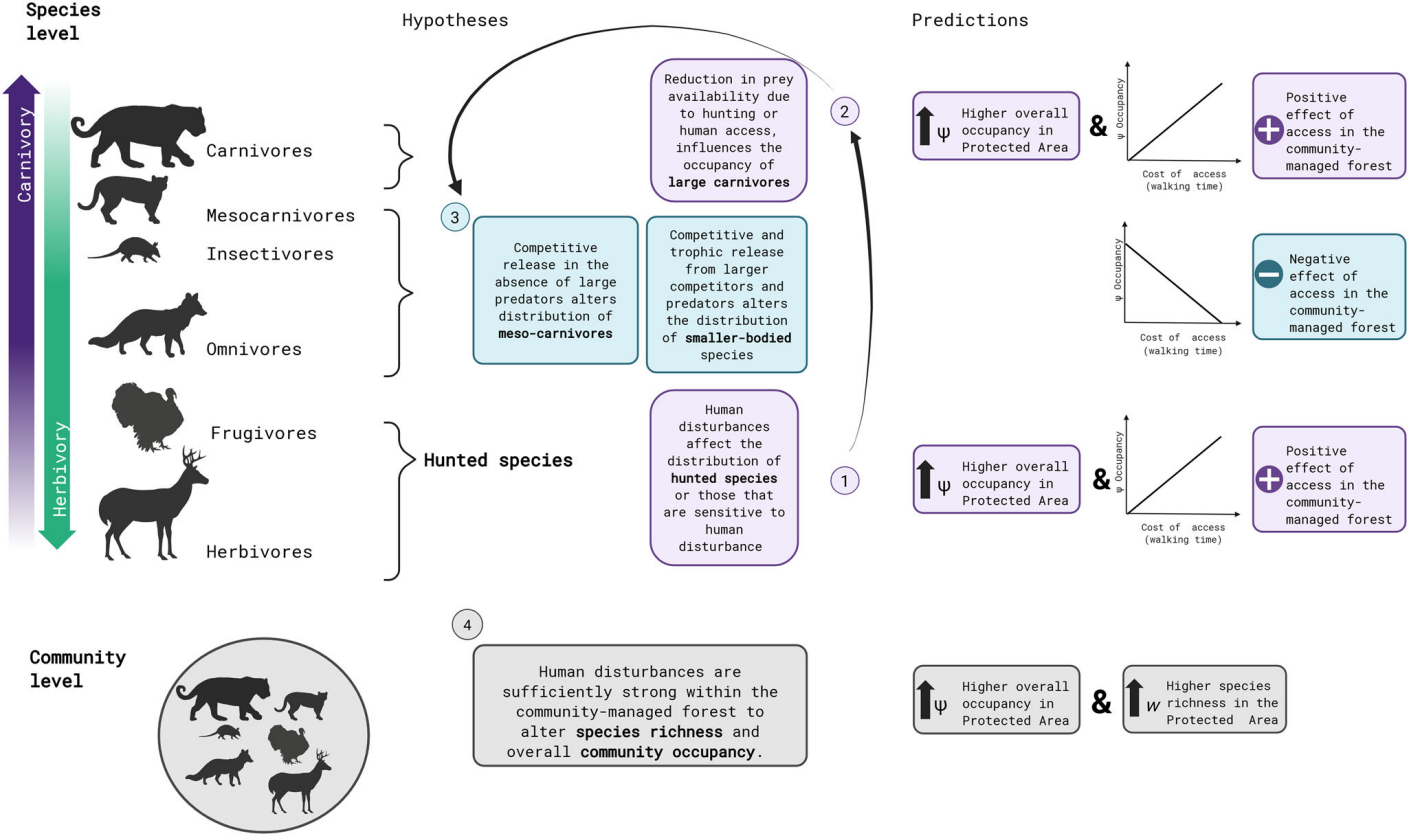
Hypotheses on and predictions about the impacts of human access on the occupancy of medium to large terrestrial vertebrates at the species and community levels in community‐managed areas and strictly protected Neotropical forests.

Because the community‐managed area is subject to greater human access due to subsistence hunting and other forest resource use, we expected differences in how species and the community responded to access to the community‐managed and protected area. We expected species occupancy rates between the 2 management schemes to differ, based on the hypothesized relationship between human disturbance and species response (Figure 1). In brief, due to the direct effect of hunting on mortality or the indirect impact of hunting and human presence on prey availability, we predicted that hunted and sensitive species and large carnivores would show higher occupancy estimates in the protected area and a more positive response to increased cost of access (i.e., higher occupancy farther from the community) in the community‐managed forest than in the protected area. In contrast, due to the combination of competitive and trophic release associated with the reduction of larger‐bodied species, we expected small and generalist herbivores, omnivores, and mesocarnivores to respond negatively to increased cost of access (i.e., higher occupancy closer to the community) in the community‐managed forest but not in the protected area. We predicted higher species richness and mean community occupancy (average occupancy across all species in the community) in the protected area, given its lower level of disturbance (Figure 1).

## METHODS

### Study area

We conducted our study in a subsistence hunting area, a national park, and the Dos Lagunas Wildlife Preserve. The hunting area was the Uaxactun community forest management unit (17°40ʹN, 89°63ʹW), which covers 835 km^2^. The strictly protected areas were north of Uaxactun: Mirador‐Rio Azul National Park (MRANP) (17°75ʹN, 89°90ʹW, 1177 km^2^) and Dos Lagunas Wildlife Preserve (17°69ʹN, 89°53ʹW; 296 km^2^). For simplicity, we refer to these 2 protected areas as MRANP. The MRANP (International Union for Conservation of Nature [IUCN] category II) was established in 1990, and Uaxactun (home to ∼800 people) started operating in 2000 and achieved FSC certification in 2001. The primary disturbances in the community‐managed forest were subsistence hunting and human presence due to low‐intensity selective logging and nontimber forest product harvesting. The more remote, formally protected area, which extends into Mexico's Calakmul Biosphere Reserve, prohibited hunting and was actively patrolled, albeit at low intensity. Access was limited to illegal border crossing from Mexico. However, smaller communities in Mexico (∼300 inhabitants) used the Mexican landscape northeast of the protected area. Although subsistence hunting occurred in the Uaxactun community management unit, residents committed to protecting species with low densities and low reproductive rates (NPV‐OMYC, 1999) prohibited hunting at waterholes and banned the sale of hunting meat to individuals outside the community and visitors to Uaxactun (NPV‐OMYC, 1999). The predominant vegetation was evergreen medium‐high lowland forest, known locally as *alto* (i.e., tall forest), and canopies were up to 30 m. A less common forest type, characterized by deciduous tree species of <15 m in areas that are seasonally inundated, is referred to as *bajo* (i.e., low forest) (Turner II et al., 2003).

**FIGURE 2:**
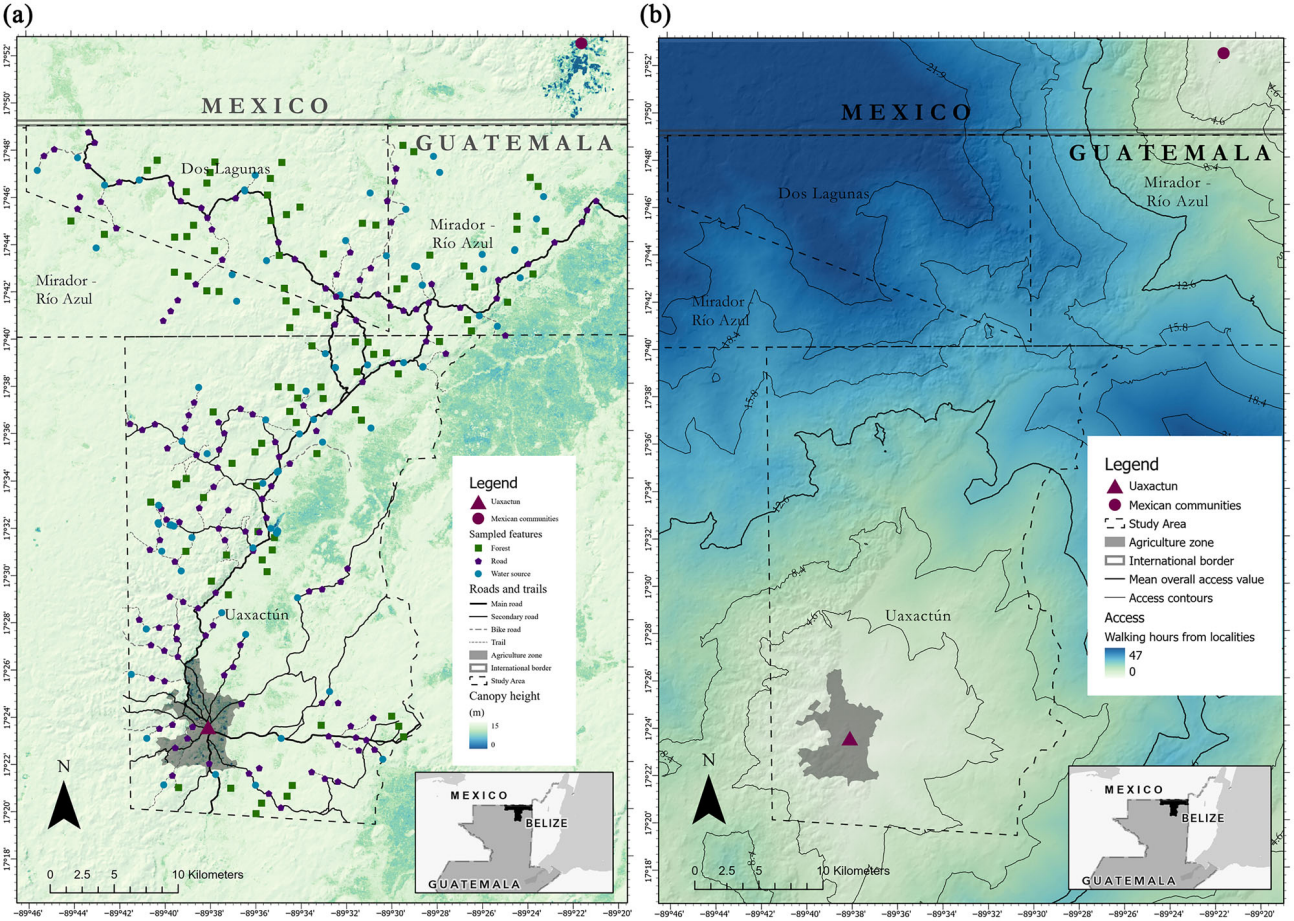
(a) Distribution of camera trap stations across the forest management unit of Uaxactun and Mirador‐Rio Azul National Park (MRANP), a protected area, relative to roads (no roads extended from Mexican communities into Guatemalan protected areas), Uaxactun, and Mexican communities (darker color shades, low canopy and seasonally inundated forests) and (b) cost of access (walking hours) from settlements to a given location in the study area (contours, location of areas with overall mean access value of 12.6 h; thick line, value at which model parameters were estimated; first and second quantile values are for camera traps in Uaxactun [4.6 and 8.4 h]; first, second, and third quantile values are for camera traps in MRANP [15.8, 18.4, and 21.9 h, respectively]).

Central to our focus on the effects of cryptic below‐canopy disturbance was the quantification of proximity to human influence. A single road connected our study areas (Figure 2a). One of the access points to MRANP was through Uaxactun. The areas designated for agriculture were within a ∼6‐km radius of Uaxactun. Subsistence hunting and *xate* palm harvest (*Chamaedorea* spp.), a forest product exported for the floristry business, were allowed throughout the management unit. Access to these resources was predominantly on foot from Uaxactun. Only a few subsistence hunters and harvesters owned motorbikes and ventured into less accessible regions. Monthly rotational xate harvest campsites were dispersed throughout the management unit and served as daily access points for xate harvesters (Balas‐McNab, 1998). Park guards and rangers traveled to and from MRANP to complete monthly rotations of guarding duties. Legal human activities in MRANP were limited to research, park management, patrolling, and sporadic tourism. The spatial distribution of these activities created a gradient ranging from areas of high human use and hunting frequency in areas near Uaxactun to places less frequently visited by tourists or park rangers in MRANP (Figure 2b).

We studied medium‐to‐large (>1 kg) avian and mammal species with terrestrial foraging strategies, including scansorial mammals, that were predicted to occur in the study region (Appendix S1). We used the IUCN Red List to identify all extant species in the Maya Forest, the Petén‐Veracruz Moist Forest, or the Yucatán Moist Forest. We excluded vultures (*Cathartiformes*) because although they forage on the ground, they spend more time in other forest strata. Our final selection included 25 mammal and 3 avian species, which we further categorized by trophic guild and hunter preference (Appendix S1).

### Camera trapping surveys

We conducted camera‐trap surveys March–June 2018 in Uaxactun and April–May 2019 in MRANP (i.e., dry season) (Appendix S2). We placed cameras on roads, at waterholes (locally *aguadas*) (aimed at the shore or trails leading to the water), and at off‐road sites to account for mammals’ differential use of habitat features (Harmsen et al., 2010) (Figure 2a). Each station had one camera trap placed approximately 45 cm from the ground. Off‐road cameras were placed on fruiting trees, wildlife trails, or topographic features.

We distributed the camera‐trapping effort by dividing the entire region into 9‐km^2^ hexagons and selected a stratified sample based on access. This allowed us to cover multiple home ranges for most of the species. Given logistical limitations, we surveyed groups of adjacent hexagons before moving to another group. Three cameras were placed in each hexagon, separated by at least 1 km. When possible, we sampled 3 different features (road, waterhole, and forest). The cameras were operational for 21–25 days. We surveyed 188 and 138 stations across 739 km^2^ (88.5%) of Uaxactun and 548 km^2^ (37%) of MRANP, respectively, for a total of 326 stations and 1287 km^2^. We used camtrapR (Niedballa et al., 2016) to sort images by species and generate detection histories of 3‐day occasions. We also used CamtrapR to report raw capture frequencies by species.

### Community occupancy model

We used a multispecies occupancy model with data augmentation (Dorazio & Royle, 2005) and a fixed species list of 28 species that occur in the area to estimate the occupancy probability of all medium and large species in our defined study area. In so doing, we quantified local species richness (Sutherland et al., 2016; Tenan et al., 2017). This hierarchical Bayesian model allowed us to determine how species‐specific occupancy and detection parameters responded to variables of interest. The model had species‐specific intercepts and slopes of detection and occupancy responses as random effects, as well as a shared distribution that defined a community‐level response. Data augmentation for unobserved species allowed us to estimate the species richness by treating occupancy as a latent variable and including a species‐specific indicator variable, *w*, to estimate community membership. The model estimated community‐level and species‐specific detection rates, occupancy rates, and the size of the terrestrial communities studied. Occupancy probability was the probability of a species using the camera trap area, not actual occupancy, especially for large species with large home ranges. We used occupancy, although the biological interpretation of our estimate is site use for some of these larger species.

At the species level, the occupancy probability of a given species k for a given site i is denoted by $\psi_{ki}$. Detection probability that species k occurs at site i on occasion j is denoted by $p_{kij}$. Both detection and occupancy are Bernoulli processes, and detection is conditional on the true, unknown, and latent occupancy state $z_{ik}$.

Occupancy probability for each species *k* for each site *i*, $\psi_{ki}$, was estimated using the following logit linear model:

(1) $$\begin{array}{ccc} \mathrm{logit}\;\left(\psi_{ki}\right) & = & \beta_{0ka}+\beta_{1ka}\times \mathrm{acces}s_{i}+\beta_{2k}\times \mathrm{canop}y_{i}\\ & & +\;\beta_{3k}\times \mathrm{EV}I_{i}+\beta_{4k}\times D2W_{i}+\beta_{5k}\times \mathrm{SLOP}E_{i}\\ & & +\;\beta_{6k}\times \mathrm{ELE}V_{i}, \end{array}$$

where *a* is area, access is the cost of access measured in walking travel time from the community to camera trap locations, canopy is canopy height, EVI is enhanced vegetation index, D2W is distance to water sources, and ELEV is elevation.

The random effects $\beta_{0ka}$ and $\beta_{1ka}$ are modeled as species‐specific random effects with an area‐specific mean: $\beta_{mka}\;∼\;\mathrm{normal}\left(\mu_{ma},\sigma_{ma}\right)$ for m∈{0,1}, whereas the coefficients $\beta_{2}\;$ to $\beta_{6}$ are species‐specific random effects that are constant between areas: $\beta_{mk}\;∼\;\mathrm{normal}\left(\mu_{m},\sigma_{m}\right)$ for m∈{2,6}. The species parameter inclusion per each site *w* is $\omega_{k.\mathrm{area}}∼\;\mathrm{beta}\left(\Omega \right)$ and indicates whether a species is part of each area's community (Uaxactun or MRANP).

To measure potential human access to a site, we calculated walking access or travel time in hours from settlements according to Weiss et al. (2018) and used additional ground‐truthed road data. Our access variable likely reflected a gradient of subsistence hunting and human presence from activities, such as xate harvest or selective timber extraction. Because we predicted contrasting responses to human access between Uaxactun and MRANP and differences in occupancy rates, we fitted a model with different intercepts and slopes for the access–occupancy relationship for each area. We also modeled the influence of forest canopy height, enhanced vegetation index, distance to water sources, slope, and elevation on mammal occupancy and richness under the intact canopy (Appendix S3).

Because species may be less elusive in protected areas, detection probability was modeled differently based on protection type. Therefore, we used area as a categorical covariate for all detection estimates. In our hierarchical analyses, the probability for each occasion, j, of a species, k, at a site, i, was defined by feature type, *f* (road, forest, or water), and for each area, *a*, separately as

(2) $$\mathrm{logit}\left(p_{ijk}\right)=\alpha_{0kaf}+\alpha_{1ka}\times \mathrm{da}y_{ij},$$

where the species and area‐specific detection random intercepts $\alpha_{0kaf}\;$ have a feature‐specific mean $\alpha_{0kaf}\;∼\;\mathrm{normal}\left(\mu_{\alpha 0f},\sigma_{\alpha 0f}\right)$, and the effect of seasonality on detection $\alpha_{1ka}$ is a species‐specific random effect slope per area $\alpha_{1ka}\;∼\;\mathrm{normal}\left(\mu_{\alpha 1},\sigma_{\alpha 1}\right)$. We used Julian days, or the number of continuous days since 1 January, for a given year to measure seasonality.

We analyzed the models with Markov chain Monte Carlo (MCMC) simulations in Jags 4.3.0 through the JagsUI package 1.5.1 (Kellner, 2015) in R (R Core Team, 2020). We generated 3 chains of 150,000 iterations each, ran 15,000 iterations as an adaptive phase, maintained a chain every 20 iterations, and obtained 22,500 posterior samples. For priors, we followed the recommendations of Guillera‐Arroita et al. (2019) and used a beta distribution (0.001, 1) for omega, a normal distribution (0, 0.2) for the mean of intercepts and slopes of all our hyperparameters, and a uniform distribution for the standard deviation *U* (0, 5) of those intercepts and slopes. We visually inspected the trace plots to assess convergence and checked that each estimated parameter's Rhat values (Brooks–Gelman–Rubin statistic) were <1.1. We evaluated the model fit by calculating the Bayesian *p* value with a Freeman–Tukey deviance statistic between our observed detection data and a simulated one from the posterior distribution of our fitted model, where we obtained a value of 0.47 (values close to 0.5 indicate a good fit) (Kery & Royle, 2016).

To communicate the results of our multispecies model, we determined the influence of access versus habitat covariates on occupancy by comparing the increase in odds ratios and occupancy probability at mean covariate values. We also estimated the maximum possible change in occupancy due to each covariate by calculating the difference between occupancy at maximum and minimum values of each covariate. Additionally, we also quantified the probability that mean occupancy was higher in the protected area than in the forest management unit by quantifying the proportion of the posterior mean occupancy with higher values in the protected area. That is, a 100% chance of higher occupancy probability in MRANP would translate into no overlap between the posterior distributions of the 2 management strategies, whereas a 70% chance of higher probability would indicate that 30% of the MRANP posterior distribution overlapped that of Uaxactun or there was a 30% chance of similar occupancy in both protection types. We classified values above 60% or below 40% as a higher or lower probability of occupancy in MRANP, respectively. Finally, for both of our study areas, we derived species richness per site and area as the sum of the estimated latent occupancy state *z* from all sites and the species parameter inclusion *w* for each area, respectively.

## RESULTS

Our 326 camera trap deployments resulted in 7984 trap nights (TN) in Uaxactun and MRANP (Appendix S2). We recorded 26 of 28 species in our terrestrial vertebrate community, with 24 species in both regions. Species differed between regions. The most frequently recorded species in both areas was the great curassow (*Crax rubra*), followed by the tapir (*Tapirus terrestris*) in the protected area and the white‐lipped peccary (*Tayassu pecari*) in Uaxactun (Appendix S4). We did not detect the jaguarundi (*Herpailurus yagouaroundi*) in MRANP or the coyote (*Canis latrans*) in Uaxactun. Species with fewer than 10 detections had wide and flat rather than informative posterior distributions for most parameters. Therefore, we do not report the occupancy probability of the coyote, jaguarundi, northern tamandua (*Tamandua mexicana*), or raccoon (*Procyon lotor*) in either area or the striped hog‐nosed skunk (*Conepatus semistriatus*) in the protected area (Appendix S4). Our camera traps surveyed a range of access values from 0.5 to 16.5 walking hours in Uaxactun and from 8.8 to 25.3 walking hours in MRANP. Upon standardization, the overall mean of our camera trap locations was 12.6 walking hours, at which all occupancy estimates are reported.

### Community and species occupancy

**FIGURE 3:**
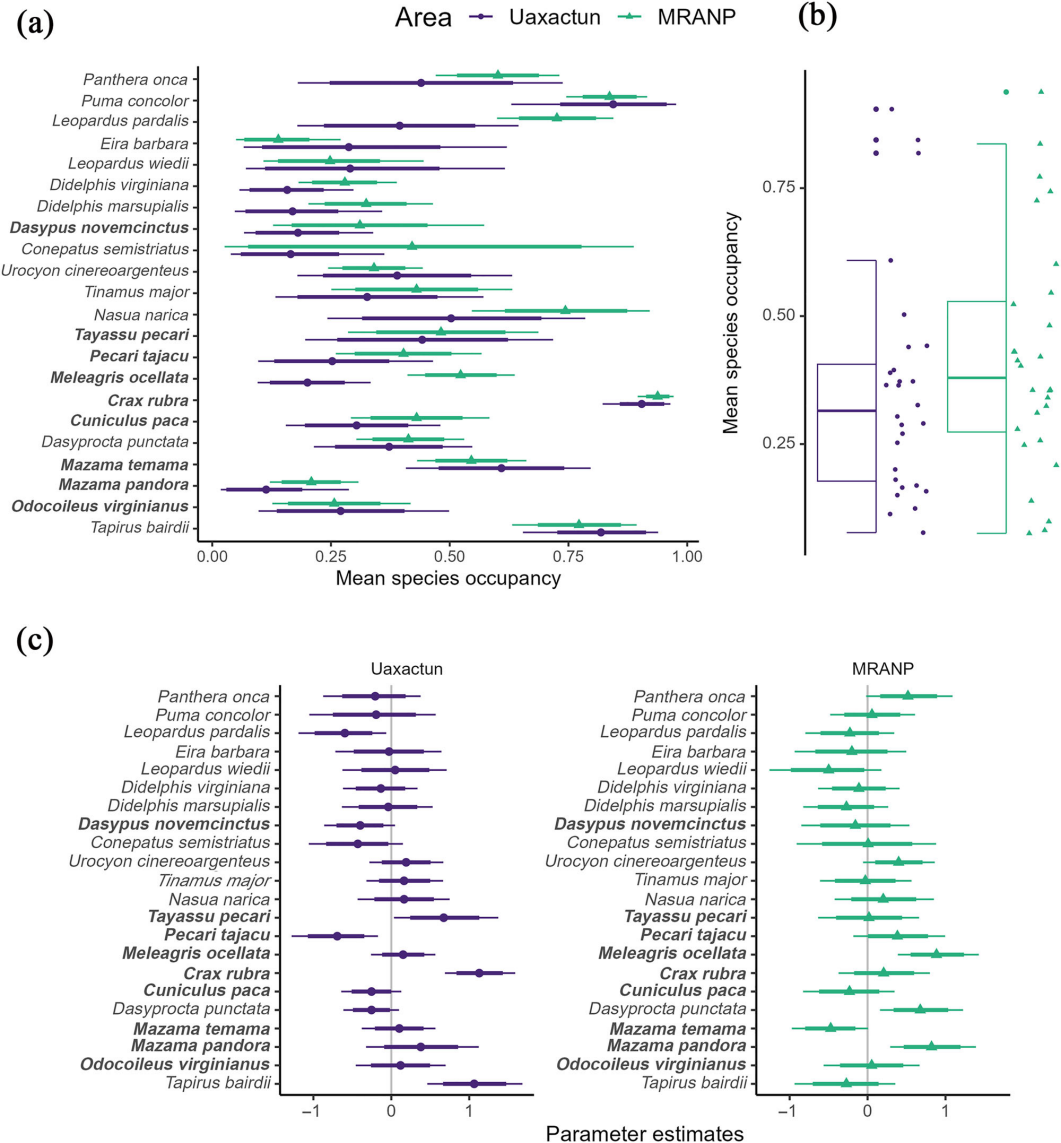
Mean (a) species and (b) overall community occupancy for Uaxactun and Mirador‐Rio Azul National Park (MRANP) and (c) estimates of the effect of human access on the occupancy probability (positive values, species with high occupancy probability in more remote or less accessible areas; whiskers, 85% and 66% Bayesian credible intervals; species ranked by trophic level and body size; bold, hunted species).

When comparing occupancy estimates between the 2 areas (Uaxactun and MRANP), we found that 60% of the species (14 of 22 species) had at least a 60% probability of estimates being higher in MRANP than in Uaxactun (Figure 3a; Appendix S5). For 6 of these species (ocellated turkey [*Meleagris ocellata*], ocelot [*Leopardus pardalis*], both opossum species [*Didelphis virginiana* and *Didelphis marsupialis*], coati [*Nasua narica*], and Yucatan brown brocket [*Mazama pandora*]), there was at least an 85% probability of higher occupancy values in MRANP. The greatest differences were for the ocellated turkey (100%) and the ocelot (97%) (Figure 3a; Appendix S5). In contrast, 4 species had a higher probability of occupancy in Uaxactun than in MRANP, which included the tayra (*Eira barbara*), the red‐brocket deer (*Mazama temama*), the Baird's tapir, and the puma (*Puma concolor*) (Appendices S5 & S6). Four hunted species had mean occupancy values below 0.35 in Uaxactun, including ocellated turkey, collared peccary (*Pecari tajacu*), paca (*Cuniculus paca*), and great tinamou (*Tinamus major*). The Yucatan brown brocket, white‐tailed deer (*Odocoileus virginianus*), and armadillo (*Dasypus novemcinctus*) had low mean occupancy values in Uaxactun and MRANP (Figure 3a; Appendix S6). At the community level, Uaxactun and MRANP had similar overall mean community occupancy estimates of 0.35 (85% Bayesian Credible Interval (BCI) 0.10–0.86) in Uaxactun and 0.42 (85% BCI 0.08–0.87) in MRANP (Figure 3b).

**FIGURE 4:**
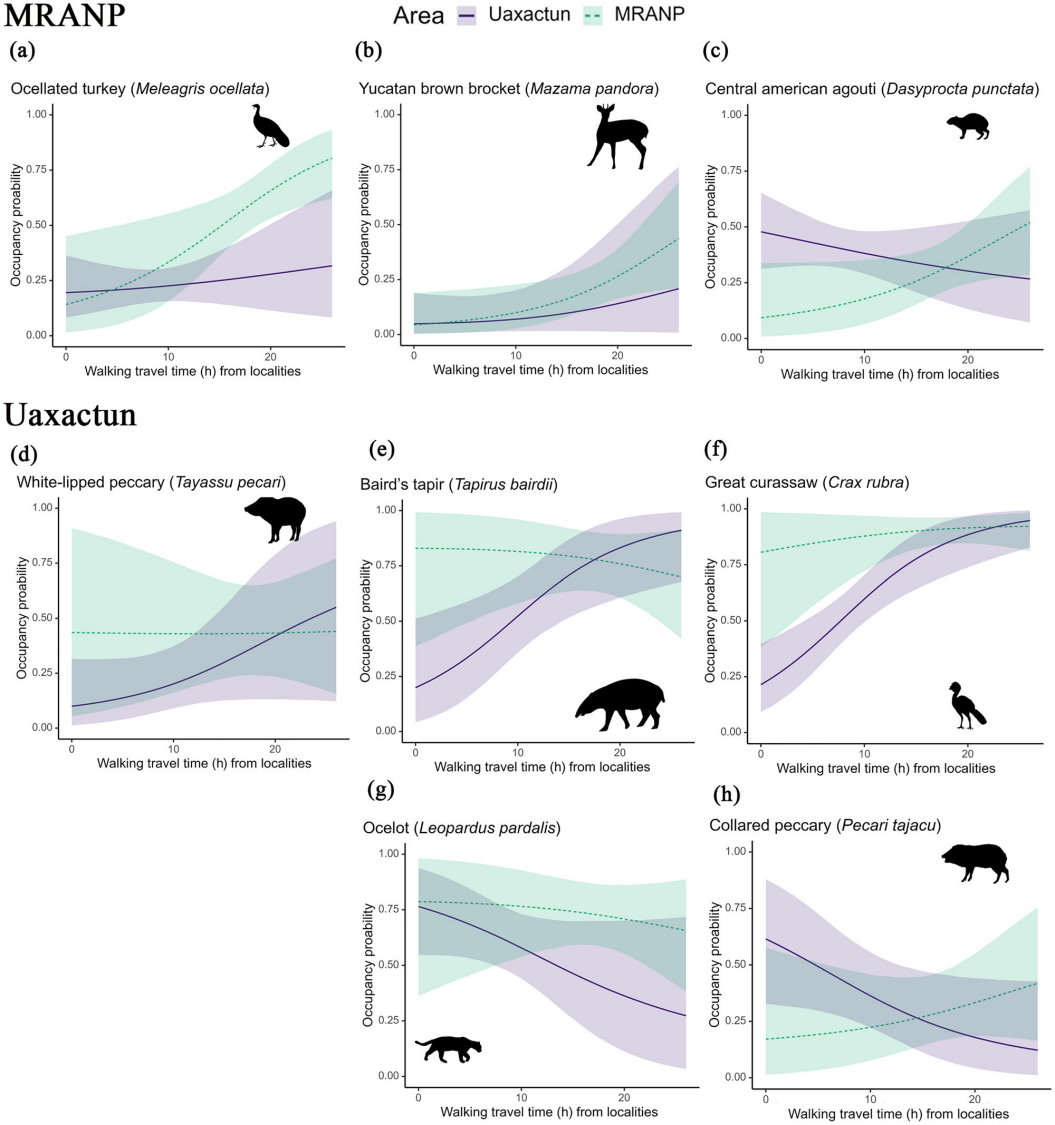
The influence of human access on the occupancy of the (a) ocellated turkey, (b) Yucatan brown brocket, and (c) agouti in Mirador‐Rio Azul National Park (MRANP) and of the (d) white‐lipped peccary (e) Baird's tapir, (f) great curassow, (g) ocelot, and (h) collared peccary in Uaxactun.

Based on species‐specific parameter estimates from our occupancy model, human access from localities influenced the occupancy of 8 out of 22 species at an 85% BCI or above (where BCI indicated the proportion of the posterior distribution greater than or less than 0) (Figure 3c). Three species responded positively to less accessible areas in the protected area (i.e., with higher travel time from communities), and 5 did so in Uaxactun (Figure 3c). Our predictions regarding how different groups of species would respond to human access were partially supported by our results. One or 2 species per trophic guild or hunting preference category exhibited responses that corresponded to our predictions. Three large or hunted species or both, the great curassow, Baird's tapir, and the white‐lipped peccary (*Tay. pecari*), had a positive association between occupancy and lower human access in Uaxactun (100%, 99%, and 94% BCI, respectively) (Figure 3c; Appendix S7). For every 6 h walked away from Uaxactun, the odds of occupancy increased by 210%, 191%, and 99% for the great curassow, the tapir, and the white‐lipped peccary, respectively (Figure 4; Appendix S7). We found the same response for 2 large and hunted species in the protected area. For every 6 h farther from Mexican communities, the odds of occupancy for the ocellated turkey and the Yucatan brown brocket increased by 145% and 130%, respectively. The Central American agouti, a small, nonhunted species, also responded positively to less accessible areas in MRANP, with the odds of occupancy increasing by 98% every 6‐h walk away from the Mexican communities (Figure 3c; Appendices S5 & S7).

In contrast, only 2 species, including one mesocarnivore and one smaller competitor, which we expected to have higher occupancy near communities (i.e., a negative slope), confirmed our expectation at 85% BCI. These species were the ocelot and the collared peccary (Figure 4). In Uaxactun, for every 6 h walked away from the community, the odds of occupancy decreased by 46% and 51% for the ocelot and collared peccary, respectively (Appendix S7) Of the species that responded to access, all but the ocelot in Uaxactun responded more strongly to access than all other habitat variables, indicating the importance of this variable in controlling distribution patterns in intact forests. For the Yucatan brown brocket, the influence of access was more important than habitat covariates in Uaxactun and in MRANP (Appendix S5).

Contrary to our predictions, the effect of human access on the occupancy of large carnivores (Figure 3c; Appendix S7) was partially supported only for jaguars (*Panthera onca*) in the protected area instead of the community‐managed forest. For the jaguar, access was also more important than the rest of the covariates in MRANP, possibly showing a response to the Maya forest edge near Mexican communities (Appendices S5 & S7). Finally, human access did not influence the overall community occupancy probability (0.04, 85% BCI −0.14 to 0.22) (Appendices S8 & S9), likely due to the high variability in positive and negative responses of individual species.

Most of the habitat covariates did not contribute as much as human access to the variation in occupancy. Although it could potentially contribute the most (Appendix S5), no habitat covariate was significant as frequently as access. From the habitat covariates, canopy height was the most important. Five species showed a probability of association with canopy height higher than 85% BCI, followed by elevation and distance to water with 3 and 2 species, respectively (Appendices S10 & S11). Canopy height had a positive influence on the overall community occupancy (0.13, 85% BCI 0.03–0.23) (Appendices S8 & S9).

Seasonality affected the detection probability of 10 species both positively and negatively (Appendices S12 & S13). Camera placement was important. Species detection probability varied across the 3 feature types (Appendices S13 & S14). Thirteen species and the overall community had higher detection probabilities at roads and waterholes than in forests (Appendices S15–S17).

### Species richness

The estimated (modal) species richness was 24 and 25 species for MRANP and Uaxactun, respectively (Appendix S18a). Mean species richness per site (i.e., camera station) did not differ between areas; the mean species estimated per site ranged from 4 to 13 in Uaxactun (mean [SD] = 7.84 [1.90]) and from 4 to 15 (9.69 [2.05]) in MRANP (Appendix S18b). There was no relationship between estimated site species richness and human access in either of the 2 areas (Appendix S18c).

## DISCUSSION

We quantified how human activities influenced the distribution of medium‐to‐large terrestrial vertebrates in continuous intact forests. Sampling across the gradient of human access and focusing on multiple features (e.g., waterholes, roads, fruit trees) allowed us to model community responses and uncover contrasting species‐specific responses. Human access cost, or the time it takes to walk from a community to the sampling sites, affected species distribution, influencing species occupancy positively for 4 hunted species out of 6 and negatively for a mesocarnivore and a smaller competitor in areas of lower accessibility. Although we did not discriminate between different human activities, we assumed that subsistence hunting is the leading cause of observed patterns due to its positive relationship with access (Benítez‐López et al., 2017). However, daily journeys to harvest forest products or engage in logging activities may also have contributed to the patterns we found. These complex responses to human access under intact forest canopy demonstrate the need for resources to conduct the difficult field work necessary to go beyond remotely sensed measures and examine what is occurring below the canopy to evaluate biodiversity outcomes in protected and other managed areas (Geldmann et al., 2019). We also believe that to evaluate biodiversity outcomes in managed areas subject to subsistence hunting and other forest resource use, examining species‐specific responses is much more informative than relying on species richness alone. Our results pertain only to one season—the dry season—and additional information from the wet season would provide a more complete picture of species response to cryptic disturbance.

Detecting and understanding these patterns at representative spatial scales is essential for all types of protection categories because they can be influenced by managed and illegal human activities. Robust evidence of conservation effectiveness is still scarce in strictly protected and managed forests (Geldmann et al., 2021; Rodrigues & Cazalis 2020). In the context of FSC community‐management initiatives, the evidence has only just begun to emerge (Burivalova et al., 2019). Our study may be the first study of mammals in an FSC management area (Matias et al., 2024). Given the remoteness of our comparator, the MRANP, our results indicated a positive but nuanced evaluation of the Uaxactun FSC‐certified community‐managed forest. We found relatively similar community metrics between Uaxactun and the strictly protected area, but at the species level, we found some differences between the 2 management categories that were driven by human access.

### Species responses

We expected that certain species would be more affected by subsistence hunting and extractive activities. Except for large carnivores, the observed patterns were consistent with our predictions, although we found impacts for fewer species than expected. Three of the largest mammalian herbivores (white‐lipped peccary, Yucatan brown brocket, and tapir) and the 2 largest ground‐dwelling birds (great curassow and ocellated turkey) had lower occupancy rates in areas closer to local communities. For 3 of these species (great curassow, white‐lipped peccary, and tapir), this effect was pronounced in Uaxactun and not evident in the protected area, as we predicted, possibly indicating higher abundances and the absence of anthropogenic pressures on these species in the protected area. However, the 2 endemic species (i.e., ocellated turkey and Yucatan brown brocket) were affected by access more strongly in the protected area.

The white‐lipped peccary and the tapir are of regional conservation concern in Central America, given the fragmented and declining distributions (Schank et al., 2020; Thornton et al., 2020). The white‐lipped peccary is preferred by hunters, which, combined with their low fecundity, social behavior, large home ranges, and reliance on water bodies (Peres et al., 1996; Reyna‐Hurtado et al., 2010), makes them highly vulnerable to persecution. Their medium overall site use rates, even in the protected area, and their response to increased human access in Uaxactun indicated their sensitivity to hunting and their overall sensitivity even in one of the most remote areas in the Maya Forest. Baird's tapir, although not a documented game species in this area (McNab et al., 2019), might be responding to a combination of habitat disturbance and human presence in the forest or at water resources, which are a scarce resource in the Maya Forest (Reyna‐Hurtado et al., 2019).

Great curassows are the most hunted species in Uaxactun (McNab et al., 2019) and had a markedly lower occupancy in areas with increased human access in Uaxactun. They appeared to be thriving in areas where subsistence hunting was less frequent. These results add evidence to the potential of curassow species to serve as bioindicators of hunting pressure, as previously proposed (Silva & Strahl, 1991). Ocellated turkeys also had lower overall occupancy in Uaxactun, which suggests a higher impact in this area, possibly because of their vocalizations and higher detectability by hunters, especially during the mating season. Contrary to the great curassow and our prediction, the ocellated turkey responded negatively to increased human access more strongly in the protected area. Potentially, ocellated turkeys’ occasional use and incursion into open habitats and agricultural lands, together with their larger home range (Gonzalez et al., 1998; Martin & Buchholz, 2018), may put them in greater contact with Mexican communities across the protected area borders than curassows. Similarly, the lower occupancy of Yucatan brown brockets near Mexican communities may indicate lower abundance due to hunting pressure across the border, whereas, in Uaxactun, this species might be naturally rare or already affected by subsistence hunting or both.

Our results provide evidence of a compensatory effect at an 85% BCI for only one small herbivore and one mesocarnivore. The collared peccary and the ocelot had higher occupancy in areas with more human access (i.e., closer to the community), and this effect was more pronounced in Uaxactun than in the protected area. Two small herbivores, one omnivore (hog‐striped skunk [*Conepatus semistriatus*]), and one insectivore (armadillo) had similar patterns in Uaxactun but with probabilities from 80% to 85%. The responses observed for the small herbivores, the Central American agouti, and (to a much lesser extent) the lowland paca have been documented in other hunted forests (Galetti et al., 2015; Williams‐Guillén et al., 2006). Ocelots also had a higher probability of using accessible areas and could be responding to a relatively higher presence of agoutis, an important food item (Suselbeek et al., 2014). The patterns observed in Uaxactun for paca, agoutis, and ocelots have also been reported in the absence of hunting following the natural crash of white‐lipped peccaries in the Amazon and forests subject to tourism with lower relative abundances of tapirs, jaguars, and peccaries, suggesting a common mechanism of trophic release (Ouboter et al., 2021; Whitworth et al., 2022).

Not all species conformed to our expectations. One large but less hunted species (collared peccaries) was more common near the community, and the white‐tailed deer was uncommon throughout the entire study area. For collared peccaries, the observed patterns could be a combination of a more generalist resource use (Barreto et al., 1997) and release from competition with white‐lipped peccaries (Ferreguetti et al., 2018), which is a highly hunted species. For the white‐tailed deer, although our results could reflect its preference for habitat that is more open or modified than the tall forest (Weber, 2008), its overall low occupancy observed in both areas could also reflect hunting pressure extending into MRANP populations. Although we did not expect a response to access for jaguars in the protected area, the observed estimates might indicate a response to forest edge on the Mexican side of MRANP. More surprisingly, jaguars and pumas did not have lower occupancy probabilities near the Uaxactun community. However, given that we report the use of space rather than occupancy for these wide‐ranging species, the lower detection probabilities in Uaxactun (Appendix S16) for jaguars and pumas may indicate more extensive home ranges (so the species is less likely to be captured twice in the same camera) and, consequently, a lower density of both cat species in Uaxactun than in the protected area. Finally, the Central American agouti (not much hunted) responded to access in MRANP. Its occupancy probabilities near Mexican communities were low, which merits further attention.

The mean species‐specific occupancy estimates allow a comparison of the Uaxactun–MRANP system (a hunting to no‐hunting gradient) with Tobler et al.’s (2018) study of an FSC‐certified managed area in the Maya Forest with reduced impact logging and minimal hunting pressure (referred to as Holmul). In our study, 7 of the 10 most preferred hunted species had higher occupancy in MRANP than Uaxactun (Appendix S5). However, the paca, white‐tailed deer, Central American agouti, and Yucatan brown brocket deer had even higher occupancy in Holmul, suggesting that the hunting impact from Uaxactun might extend into MRANP for these 4 species or that hunting in Mexican communities is affecting agoutis and Yucatan brown brocket populations in the protected area. Despite the structural differences in the occupancy models, this comparison provides preliminary insights into the status of hunted species in our study area and an initial hypothesis of affected populations beyond the hunted area of Uaxactun. For these 4 species, but especially for the white‐tailed deer, the impacts of hunting might be more important and affect both areas similarly (no differences in mean occupancy values) than impacts from reduced‐impact logging.

The inferred potential differences in hunted species’ occupancy metrics between management strategies led us to hypothesize that source–sink dynamics regulate species’ responses to hunting and that spatial refuges (nonhunted areas, protected areas, or remote parts of community‐managed areas) are important for sustaining hunted wildlife populations (Antunes et al., 2016).

### Community responses

Despite the observed occupancy differences between areas, our results suggest that the community‐managed forest of Uaxactun, with remote areas and limited human access, is large enough (835 km^2^) to sustain a mostly intact wildlife community with species richness and overall occupancy similar to the protected area (Appendix S18; Figure 3b). At the community level, MRANP and Uaxactun did not differ in these metrics, indicating a neutral biodiversity outcome for the community‐managed forest of Uaxactun (Burivalova et al., 2019). Given that MRANP is one of the most remote areas of the Maya Biosphere Reserve, our evaluation of the conservation benefit of the community‐managed area was conservative. In contrast, many evaluations of forest management strategies often compare areas under forest management with sites undergoing more intense extraction pressure (Zwerts et al., 2024), where it is easier to find a positive outcome. Here, the combination of relatively low human population density, large forest concession area, and remote areas free from hunting pressure likely contributed to a neutral community change relative to a strict and remote protected area.

### Factors confounded with type of protection

The differences in predicted species responses to access between conservation strategies are potentially confounded by remoteness. The west side of MRANP and its surrounding area in Mexico, the core area of Calakmul Biosphere Reserve, are some of the most isolated regions in each country. This isolation provides natural protection from human pressures, making MRANP an ideal reference site for sustaining undisturbed wildlife populations. At the same time, the ability of governments to protect these transborder areas is limited, and the remoteness makes these areas more expensive and difficult to protect. Many of the strictly protected areas in the MBR are contiguous with rural areas and lack strong protection regimes, leaving them susceptible to illegal wildlife hunting, colonization, and deforestation, as seen in Laguna del Tigre National Park (Blackman, 2015; Devine et al., 2020). Thus, the effect of the protected area we identified must be interpreted cautiously because it might not be generalizable to other protected areas in the MBR.

### Management implications

Although we did not examine changes in the social and economic aspects of the community of Uaxactun or current levels of subsistence hunting (van Vliet et al., 2015) or compare patterns observed with counterfactual scenarios (Pressey et al., 2021), the observed species‐specific spatial patterns provided metrics for biodiversity outcome monitoring and reporting for Uaxactun and MRANP (Visconti et al., 2019). The neutral biodiversity outcomes we found for community‐managed forests relative to our reference site are considered positive outcomes by Visconti et al. (2019) because they are “at least” at values similar to the values of the reference site. However, these outcomes were aggregated for species richness and overall community occupancy rather than species traits or individual focal species. At the species level, only the ocellated turkey had no overlapping occupancy metrics between the 2 areas at a given access value (Figure 3a; Appendix S5). The fact that only one species had a 100% higher probability of occupancy in the protected area (Appendix S5), in the face of contrasting management strategies for resource utilization and subsistence hunting, could be considered a qualitative success for the FSC‐related biodiversity goals (Polisar et al., 2017) of the community‐managed forest. Because our study period covered only the dry season, it is necessary to determine how these patterns change during the rainy season, when movement patterns of species and people may change substantially. However, our results can start the conversation within the Uaxactun community for prioritizing research on hunted species, setting conservation goals, and improving the state of affected species, such as the ocellated turkey. The impact of management efforts can be monitored through mean species occupancy for species of high conservation value (FSC, 2023; Polisar et al., 2017), such as the endangered white‐lipped peccary, tapir, and jaguar (CONAP, 2009), and endemic species (ocellated turkey and Yucatan brown brocket deer) to provide data on the efficacy of the biodiversity conservation mandates of FSC.

We suggest community managers continue efforts to provide alternative sustainable livelihoods to reduce people's reliance on subsistence hunting for income (McNab et al., 2019). Such efforts have already been, to some extent, successful because there has been a decline in active hunters in Uaxactun over time (McNab et al., 2019). Additionally, it is recommended that patrolling and control measures in Uaxactun and MRANP continue to prevent illegal activities that are an ongoing threat to the Mayan Forest (Radwin, 2023). Through continued efforts by communities and partner institutions, biodiversity in the Maya Forest (and other important Neotropical forests) can be conserved effectively through a spatial mosaic of linked FSC‐certified community‐managed and protected areas. Our results showed that both types of management regimes can contribute to the conservation of wildlife populations across a vast multiactor landscape.

## Supporting information



Supporting Information
