# Supplementary material for: Use of species’ responses to cryptic anthropogenic disturbances for monitoring biodiversity outcomes in tropical forests
Source: Conserv Biol. 2025 Oct 10;40(2):e70159. doi: 10.1111/cobi.70159 (PMC13036302; doi:10.1111/cobi.70159)
Supplement: Supplementary file 1 — Supporting Information [file COBI-40-e70159-s001.docx]

**Appendix. Supplementary material**

Appendix S1. List of species registered during the camera trap surveys. **Included** refers to whether the species was part of our defined community. When we did not include a species, it was either because it had a body mass < 1kg (a) or because it was an arboreal species (b). **ICUN** refers to the threat level according to the International Union for Conservation of Nature. LC = Least concern, DD = Data deficient, NT = Near threatened, VU = Vulnerable, EN= Endangered. **Hunting preference** refers to Uaxactun hunters' preferences for different species, with four being the most preferred and one being non-hunted. We calculated the species´ trophic level according to (Bogoni et al., 2019), by multiplying the proportion of diet items, as described by (Wilman et al., 2014), by a factor of one to seven from herbaceous items to endothermic prey, respectively.

| **Scientific name** | **English name** | **Included** | **IUCN** | **Hunting**  **preference** | **Biomass**  **(kg)** | **Trophic**  **level** | **Diet** |
| --- | --- | --- | --- | --- | --- | --- | --- |
| **DIDELPHIDAE** |  |  |  |  |  |  |  |
| *Metachirus nudicaudatus* | Brown four-eyed opossum | No (a) | LC | 1 | 0.38 | 4.2 | Mesocarnivore |
| *Philander opossum* | Gray Four-eyed Opossum | No (a) | LC | 1 | 0.59 | 4.7 | Mesocarnivore |
| *Didelphis marsupialis* | Common opossum | Yes | LC | 1 | 1.15 | 4.6 | Mesocarnivore |
| *Didelphis virginiana* | Virginia opossum | Yes | LC | 1 | 1.41 | 4.6 | Mesocarnivore |
| **MYRMECOPHAGIDAE** |  |  |  |  |  |  |  |
| *Tamandua mexicana* | Northern tamandua | Yes | LC | 1 | 4.19 | 5 | Insectivore |
| **DASYPODIDAE** |  |  |  |  |  |  |  |
| *Dasypus novemcinctus* | Nine-banded armadillo | Yes | LC | 3 | 3.95 | 4 | Insectivore |
| **CANIDAE** |  |  |  |  |  |  |  |
| *Urocyon cinereoargenteus* | Gray fox | Yes | LC | 1 | 4.22 | 3.4 | Frugivore |
| *Canis latrans* | Coyote | Yes | LC | 1 | 11.05 | 7 | Mesocarnivore |
| **PROCYONIDAE** |  |  |  |  |  |  |  |
| *Procyon lotor* | Raccon | Yes | LC | 1 | 5.08 | 3.8 | Frugivore |
| *Nasua narica* | Coati | Yes | LC | 2 | 3.75 | 3 | Frugivore |
| **MUSTELIDAE** |  |  |  |  |  |  |  |
| *Eira barbara* | Tayra | Yes | LC | 1 | 4.13 | 5.6 | Mesocarnivore |
| **MEPHITIDAE** |  |  |  |  |  |  |  |
| *Conepatus semistriatus* | Striped hog-nosed skunk | Yes | LC | 1 | 1.60 | 4 | Insectivore |
| **FELIDAE** |  |  |  |  |  |  |  |
| *Herpailurus yagouaroundi* | Jagouaroundi | Yes | LC | 1 | 7.00 | 5.4 | Mesocarnivore |
| *Leopardus wiedii* | Margay | Yes | NT | 1 | 3.60 | 5.2 | Mesocarnivore |
| *Leopardus pardalis* | Ocelot | Yes | LC | 1 | 10.50 | 6 | Mesocarnivore |
| *Puma concolor* | Cougar | Yes | LC | 1 | 48.00 | 7 | Apex carnivore |
| *Panthera onca* | Jaguar | Yes | NT | 1 | 81.15 | 7 | Apex carnivore |
| **TAPIRIDAE** |  |  |  |  |  |  |  |
| *Tapirus bairdii* | Baird's tapir | Yes | EN | 1 | 300.00 | 1 | Herbivore |
| **TAYASSUIDAE** |  |  |  |  |  |  |  |
| *Pecari tajacu* | Collared peccary | Yes | LC | 3 | 20.50 | 2.4 | Frugivore |
| *Tayassu pecari* | White-lipped peccary | Yes | VU | 4 | 31.80 | 2.7 | Frugivore |
| **CERVIDAE** |  |  |  |  |  |  |  |
| *Mazama temama* | Central american Red-brocket | Yes | DD | 4 | 23.00 | 1.4 | Herbivore |
| *Mazama pandora* | Yucatan brown brocket | Yes | VU | 4 | 20.55 | 1.4 | Herbivore |
| *Odocoileus virginianus* | White-tailed deer | Yes | LC | 4 | 65.32 | 1.2 | Herbivore |
| **DASYPROCTIDAE** |  |  |  |  |  |  |  |
| *Dasyprocta punctata* | Central american agouti | Yes | LC | 2 | 2.23 | 1.7 | Frugivore |
| **CUNICULIDAE** |  |  |  |  |  |  |  |
| *Cuniculus paca* | Lowland paca | Yes | LC | 4 | 9.00 | 1.8 | Frugivore |
| **TINAMIDAE** |  |  |  |  |  |  |  |
| *Crypturellus boucardi* | Slaty-Breasted Tinamou | No (a) | LC | 2 | 0.44 | 2.8 | Frugivore |
| *Crypturellus cinnamomeus* | Thicket Tinamou | No (a) | LC | 2 | 0.42 | 3.3 | Frugivore |
| *Tinamus major* | Great tinamou | Yes | NT | 2 | 1.03 | 3.1 | Frugivore |
| **CRACIDAE** |  |  |  |  |  |  |  |
| *Crax rubra* | Great curassaw | Yes | VU | 4 | 4.13 | 2.1 | Frugivore |
| *Ortalis vetula* | Plain Chachalaca | No (a,b) | LC | 2 | 0.56 | 1.6 | Frugivore |
| *Penelope purpuracens* | Crested Guan | No (b) | NT | 3 | 2.06 | 2 | Frugivore |
| **PHASIANIDAE** |  |  |  |  |  |  |  |
| *Meleagris ocellata* | Ocellated turkey | Yes | NT | 3 | 5.53 | 2.4 | Frugivore |
| **ODONTOPHORIDAE** |  |  |  |  |  |  |  |
| *Odontophorus guttatus* | Spotted Wood-Quail | No (a) | LC | 1 | 0.30 | 2.2 | Frugivore |

REFERENCES

Bogoni, J. A., Pires, J. S. R., Graipel, M. E., Peroni, N., & Peres, C. A. (2019). Wish you were here: How defaunated is the Atlantic Forest biome of its medium‐ to large‐bodied mammal fauna? PLoS ONE, 13(9), Article e0204515.

Wilman, H., Belmaker, J., Simpson, J., de la Rosa, C., Rivadeneira, M. M., & Jetz, W. (2014). EltonTraits 1.0: Species‐level foraging attributes of the world's birds and mammals. Ecology, 95(7), 2027–2027.

Appendix S2. Camera trap survey effort for the dry season surveys and its distribution across areas and years

| **Area** | **Sampling period** | **Year** | **Stations** | **Sampled area (Km^2^)** | **Trap nights** |
| --- | --- | --- | --- | --- | --- |
| **Uaxactun** | April 3-June 30 | 2018 | **188** | **739** | **4336** |
| Forest |  |  | 47 |  |  |
| Water source |  |  | 53 |  |  |
| Road |  |  | 88 |  |  |
| **Protected Area** | March 6 -June 2 | 2019 | **138** | **548** | **3637** |
| Forest |  |  | 49 |  |  |
| Water source |  |  | 32 |  |  |
| Road |  |  | 57 |  |  |
| **Total** |  |  | **326** | **1287** | **7984** |

Appendix S3. Source of covariates information

We used canopy height and cumulative EVI to distinguish between Bajo forests and forests present in elevated terrain. The canopy height measure was obtained from the Global Ecosystems Dynamic Initiative lidar project (<https://glad.umd.edu/dataset/gedi/>) (Potapov et al., 2021). To consider the EVI variability for a given location during a year, we used the median of the cumulative EVI MODIS-derived data, calculated from 2003 to 2014 and available from <http://silvis.forest.wisc.edu/data/dhis/> (Hobi et al., 2017). The cumulative EVI summarizes all available EVI readings for a single year and is one of the three dynamic indices used to describe annual vegetation seasonality. We tested the performance of these three indices by selecting random points per forest type and visual inspection of their distributions. The cumulative EVI was the measure that best distinguished between *bajos* and evergreen forests from the three available indices. Distance to water sources was generated by assessing how far away any given point on the landscape was from the nearest aguada (waterhole), river, or lake. The information on aguadas distribution was obtained from CEMEC (the Spanish acronym for the Guatemalan Monitoring and Evaluation Center) and CONABIO (the Spanish acronym for the Mexican National Commission for Knowledge and Use of Biodiversity). These two datasets were derived from visual inspection of high-resolution images followed by verification in the field whenever possible. With this procedure, sibales (larger waterbodies than aguadas) can be mapped, while aguadas are easily missed, given their small size relative to broad tree crowns or canopy cover. We supplemented these aguadas points with our GPS coordinates from the field. After producing our covariates, we standardized and corroborated that there was no collinearity among them before their inclusion in the analysis.

REFERENCES

Hobi, M. L., Dubinin, M., Graham, C. H., Coops, N. C., Clayton, M. K., Pidgeon, A. M., & Radeloff, V. C. (2017).

Potapov, P., Li, X., Hernandez‐Serna, A., Tyukavina, A., Hansen, M. C., Kommareddy, A., Pickens, A., Turubanova, S., Tang, H., Silva, C. E., Armston, J., Dubayah, R., Blair, J. B., & Hofton, M. (2021). Mapping global forest canopy height through integration of GEDI and Landsat data. Remote Sensing of Environment, 253, Article 112165.

Appendix S4. List of species registered, number of records, and capture frequency per species during the camera trap surveys. **CF** = Capture frequency is the total number of records per 1000 trap nights for each study area. Stations refer to the number of stations where the species was recorded. Species are organized first by trophic level and then by biomass whenever two species have the same trophic level. Species between 2.4 and four can also be considered as omnivores. We maintain this order to report in all graphs.

| **Diet &** |  |  |  | **Uaxactun** | | | **Protected Area** | | |
| --- | --- | --- | --- | --- | --- | --- | --- | --- | --- |
| **Scientific name** | **English name** | **Trophic level** | **Biomass (kg)** | **Records** | **CF** | **Stations** | **Records** | **CF** | **Stations** |
| **Apex carnivore** |  |  |  |  |  |  |  |  |  |
| *Panthera onca* | Jaguar | 7.0 | 81.15 | 68 | 15.68 | 38 | 444 | 122.08 | 53 |
| *Puma concolor* | Cougar | 7.0 | 48.00 | 137 | 31.60 | 77 | 324 | 89.08 | 77 |
| **Mesocarnivore** |  |  |  |  |  |  |  |  |  |
| *Canis latrans* | Coyote | 7.0 | 11.05 | 0 | 0.00 | 0 | 3 | 0.82 | 3 |
| *Leopardus pardalis* | Ocelot | 6.0 | 10.50 | 175 | 40.36 | 73 | 182 | 50.04 | 61 |
| *Eira barbara* | Tayra | 5.6 | 4.13 | 10 | 2.31 | 9 | 9 | 2.47 | 7 |
| *Herpailurus yagouaroundi* | Jagouaroundi | 5.4 | 7.00 | 6 | 1.38 | 5 | 0 | 0.00 | 0 |
| *Leopardus wiedii* | Margay | 5.2 | 3.60 | 9 | 2.08 | 9 | 11 | 3.02 | 9 |
| *Tamandua mexicana* | Northern tamandua | 5.0 | 4.19 | 4 | 0.92 | 4 | 2 | 0.55 | 2 |
| *Philander opossum* | Gray Four-eyed Opossum | 4.7 | 0.59 | 9 | 2.08 | 4 | 12 | 3.30 | 4 |
| *Didelphis marsupialis* | Common opossum | 4.6 | 1.15 | 27 | 6.23 | 14 | 63 | 17.32 | 28 |
| *Didelphis virginiana* | Virginia opossum | 4.6 | 1.41 | 66 | 15.22 | 26 | 256 | 70.39 | 28 |
| **Insectivore** |  |  |  |  |  |  |  |  |  |
| *Metachirus nudicaudatus* | Brown four-eyed opossum | 4.2 | 0.38 | 1 | 0.23 | 1 | 2 | 0.55 | 1 |
| *Dasypus novemcinctus* | Nine-banded armadillo | 4.0 | 3.95 | 81 | 18.68 | 30 | 13 | 3.57 | 9 |
| *Conepatus semistriatus* | Striped hog-nosed skunk | 4.0 | 1.60 | 24 | 5.54 | 15 | 1 | 0.27 | 1 |
| **Frugivore** |  |  |  |  |  |  |  |  |  |
| *Procyon lotor* | Raccoon | 3.8 | 5.08 | 5 | 1.15 | 4 | 2 | 0.55 | 2 |
| *Urocyon cinereoargenteus* | Gray fox | 3.4 | 4.22 | 119 | 27.44 | 36 | 440 | 120.98 | 53 |
| *Crypturellus cinnamomeus* | Thicket Tinamou | 3.3 | 0.42 | 8 | 1.85 | 5 | 8 | 2.20 | 5 |
| *Tinamus major* | Great tinamou | 3.1 | 1.03 | 87 | 20.06 | 27 | 326 | 89.63 | 32 |
| *Nasua narica* | Coati | 3.0 | 3.75 | 56 | 12.92 | 40 | 181 | 49.77 | 61 |
| *Crypturellus boucardi* | Slaty-Breasted Tinamou | 2.8 | 0.44 | 6 | 1.38 | 4 | 4 | 1.10 | 3 |
| *Tayassu pecari* | White-lipped peccary | 2.7 | 31.80 | 533 | 122.92 | 14 | 1262 | 346.99 | 20 |
| *Pecari tajacu* | Collared peccary | 2.4 | 20.50 | 115 | 26.52 | 37 | 94 | 25.85 | 27 |
| *Meleagris ocellata* | Ocellated turkey | 2.4 | 5.53 | 257 | 59.27 | 39 | 606 | 166.62 | 72 |
| *Odontophorus guttatus* | Spotted Wood-Quail | 2.2 | 0.30 | 1 | 0.23 | 1 | 1 | 0.27 | 1 |
| *Crax rubra* | Great curassow | 2.1 | 4.13 | 722 | 166.51 | 86 | 3096 | 851.25 | 127 |
| *Penelope purpuracens* | Crested Guan | 2.0 | 2.06 | 21 | 4.84 | 11 | 105 | 28.87 | 17 |
| *Cuniculus paca* | Lowland paca | 1.8 | 9.00 | 210 | 48.43 | 50 | 75 | 20.62 | 28 |
| *Dasyprocta punctata* | Central American agouti | 1.7 | 2.23 | 341 | 78.64 | 63 | 109 | 29.97 | 38 |
| *Ortalis vetula* | Plain Chachalaca | 1.6 | 0.56 | 4 | 0.92 | 4 | 34 | 9.35 | 12 |
| **Herbivore** |  |  |  |  |  |  |  |  |  |
| *Mazama temama* | Central American Red-brocket | 1.4 | 23.00 | 192 | 44.28 | 64 | 244 | 67.09 | 56 |
| *Mazama pandora* | Yucatan brown brocket | 1.4 | 20.55 | 5 | 1.15 | 3 | 107 | 29.42 | 27 |
| *Odocoileus virginianus* | White-tailed deer | 1.2 | 65.32 | 75 | 17.30 | 22 | 129 | 35.47 | 19 |
| *Tapirus bairdii* | Baird's tapir | 1.0 | 300.00 | 190 | 43.82 | 43 | 1828 | 502.61 | 68 |

Appendix S5. Occupancy metrics for Uaxactun and MRANP. We show a graphic representation of the mean occupancy probability per species per area (ranging from 0 to 1, vertical dashed line set to 0.5). The probability of the mean occupancy point estimate being higher in MRANP compared to the forest management unit^[[1]](#footnote-1)^, refers to the proportion of the posterior distribution’ draws higher for the protected area. The maximum potential influence on mu.psi, refers to the variable with the higher influence on occupancy probability, evaluated by the difference in occupancy at the maximum and minimum covariate values of our data set.


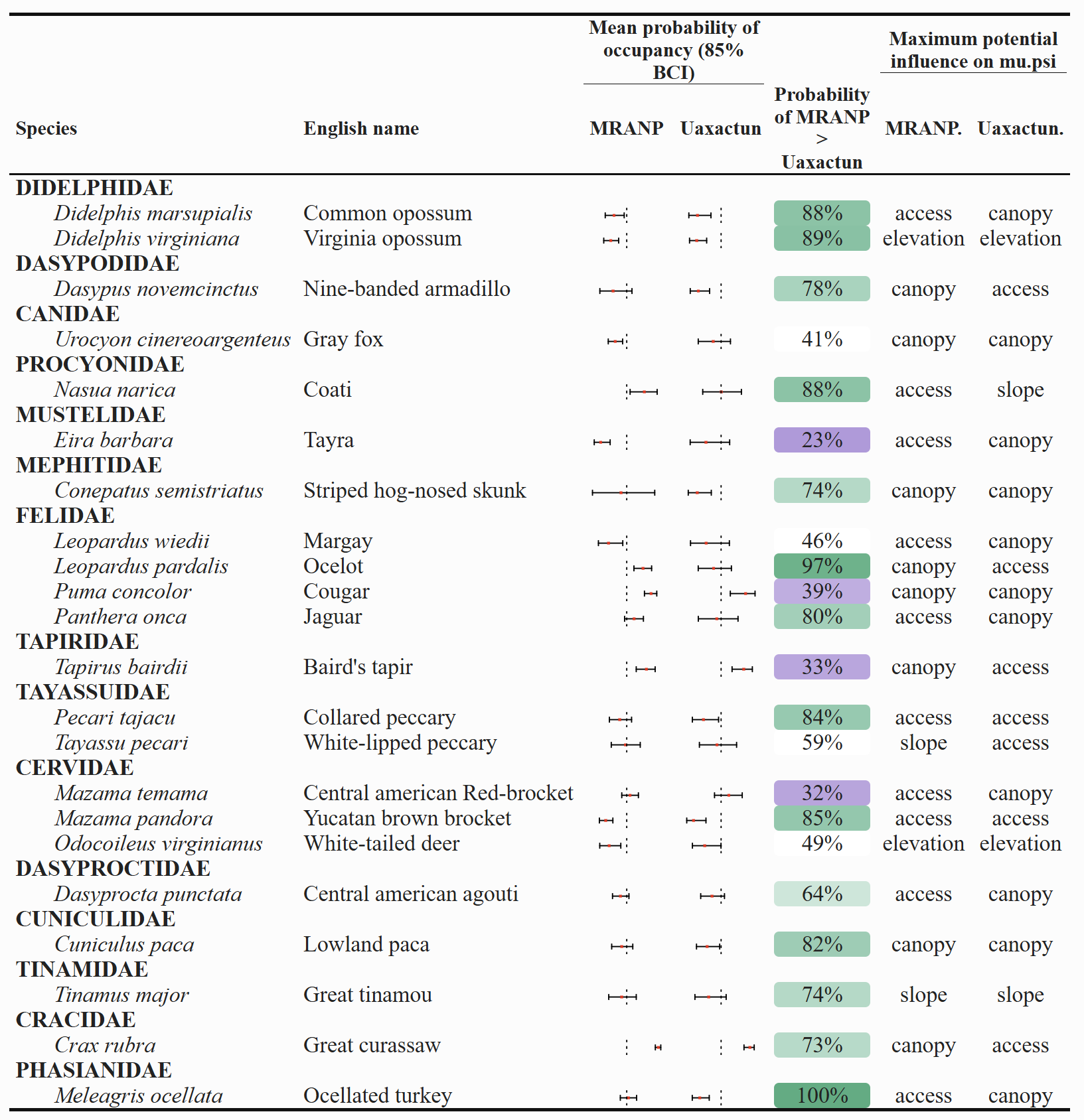


Appendix S6. Overall mean occupancy estimates per species per area and its 85% Bayesian Credible Interval.

|  | | **Mean probability of occupancy (85% BCI)** | |
| --- | --- | --- | --- |
| **Species** | **English name** | **MRANP** | **Uaxactun** |
| **DIDELPHIDAE** |  |  |  |
| *Didelphis marsupialis* | Common opossum | 0.32 (0.2-0.46) | 0.17 (0.05-0.36) |
| *Didelphis virginiana* | Virginia opossum | 0.28 (0.18-0.39) | 0.16 (0.06-0.3) |
| **DASYPODIDAE** |  |  |  |
| *Dasypus novemcinctus* | Nine-banded armadillo | 0.31 (0.13-0.57) | 0.18 (0.07-0.34) |
| **CANIDAE** |  |  |  |
| *Urocyon cinereoargenteus* | Gray fox | 0.34 (0.24-0.44) | 0.39 (0.18-0.63) |
| **PROCYONIDAE** |  |  |  |
| *Nasua narica* | Coati | 0.74 (0.55-0.92) | 0.5 (0.24-0.79) |
| **MUSTELIDAE** |  |  |  |
| *Eira barbara* | Tayra | 0.14 (0.05-0.27) | 0.29 (0.07-0.62) |
| **MEPHITIDAE** |  |  |  |
| *Conepatus semistriatus* | Striped hog-nosed skunk | 0.42 (0.03-0.89) | 0.17 (0.04-0.36) |
| **FELIDAE** |  |  |  |
| *Leopardus wiedii* | Margay | 0.25 (0.11-0.45) | 0.29 (0.07-0.62) |
| *Leopardus pardalis* | Ocelot | 0.73 (0.6-0.84) | 0.39 (0.18-0.64) |
| *Puma concolor* | Cougar | 0.84 (0.75-0.92) | 0.84 (0.63-0.98) |
| *Panthera onca* | Jaguar | 0.6 (0.47-0.73) | 0.44 (0.18-0.74) |
| **TAPIRIDAE** |  |  |  |
| *Tapirus bairdii* | Baird's tapir | 0.77 (0.63-0.89) | 0.82 (0.65-0.94) |
| **TAYASSUIDAE** |  |  |  |
| *Pecari tajacu* | Collared peccary | 0.4 (0.26-0.57) | 0.25 (0.1-0.46) |
| *Tayassu pecari* | White-lipped peccary | 0.48 (0.29-0.69) | 0.44 (0.2-0.72) |
| **CERVIDAE** |  |  |  |
| *Mazama temama* | Central american Red-brocket | 0.55 (0.43-0.66) | 0.61 (0.41-0.8) |
| *Mazama pandora* | Yucatan brown brocket | 0.21 (0.12-0.31) | 0.11 (0.02-0.29) |
| *Odocoileus virginianus* | White-tailed deer | 0.26 (0.13-0.42) | 0.27 (0.1-0.5) |
| **DASYPROCTIDAE** |  |  |  |
| *Dasyprocta punctata* | Central american agouti | 0.41 (0.3-0.53) | 0.37 (0.21-0.55) |
| **CUNICULIDAE** |  |  |  |
| *Cuniculus paca* | Lowland paca | 0.43 (0.29-0.58) | 0.3 (0.15-0.48) |
| **TINAMIDAE** |  |  |  |
| *Tinamus major* | Great tinamou | 0.43 (0.25-0.63) | 0.33 (0.13-0.57) |
| **CRACIDAE** |  |  |  |
| *Crax rubra* | Great curassaw | 0.94 (0.9-0.97) | 0.9 (0.82-0.96) |
| **PHASIANIDAE** |  |  |  |
| *Meleagris ocellata* | Ocellated turkey | 0.52 (0.41-0.64) | 0.2 (0.1-0.33) |

Appendix S7. Summary output for occupancy covariates estimates per species and area. Mean (and standard deviation) point estimates with 85% Bayesian Credible Interval (BCI). Probability indicates the percent of the posterior distribution that does not overlap zero 0. Odds ratios increase refers to the percentage of increase in the odds of occupancy per every unit of each (standardized) covariate. Probability change indicates the same, but on the probability scale.


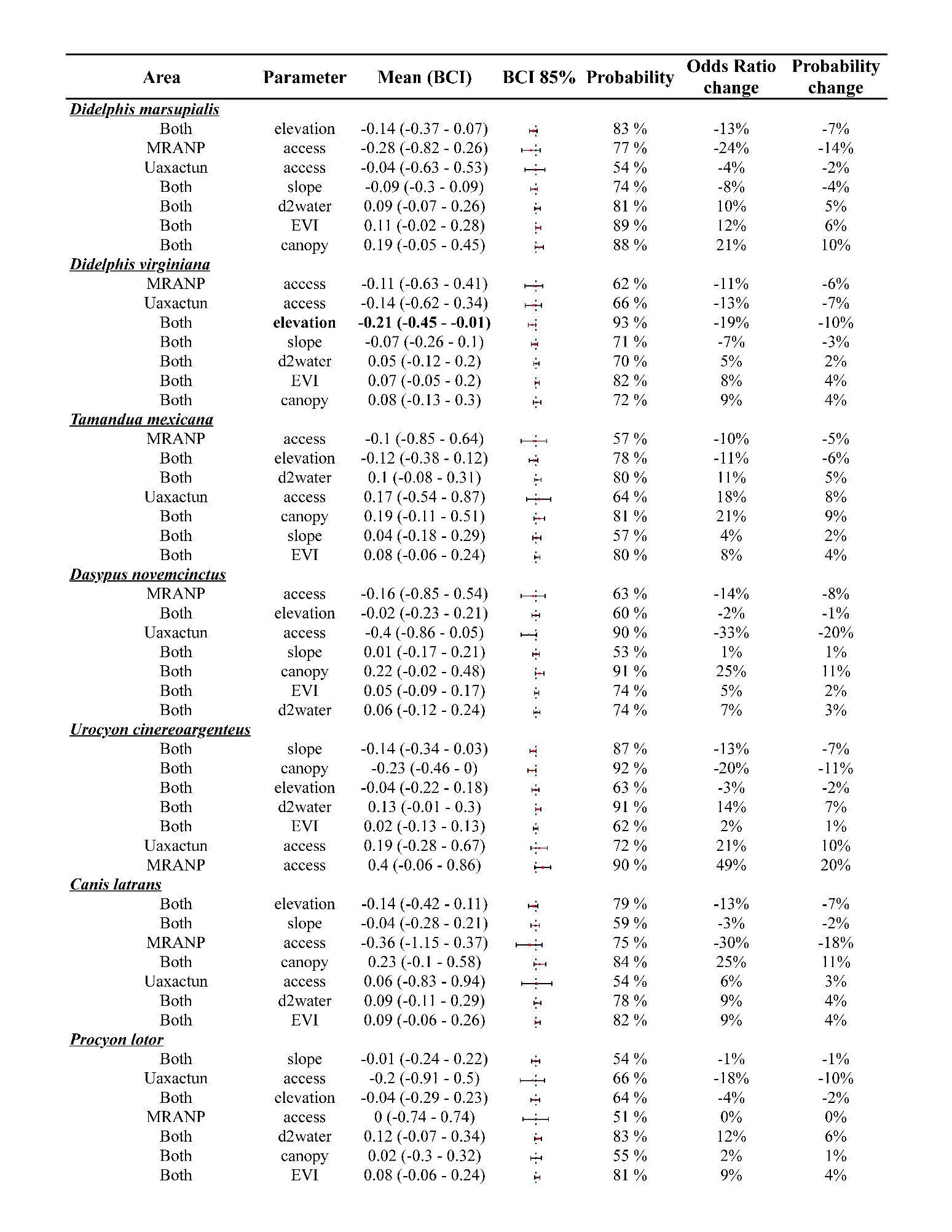

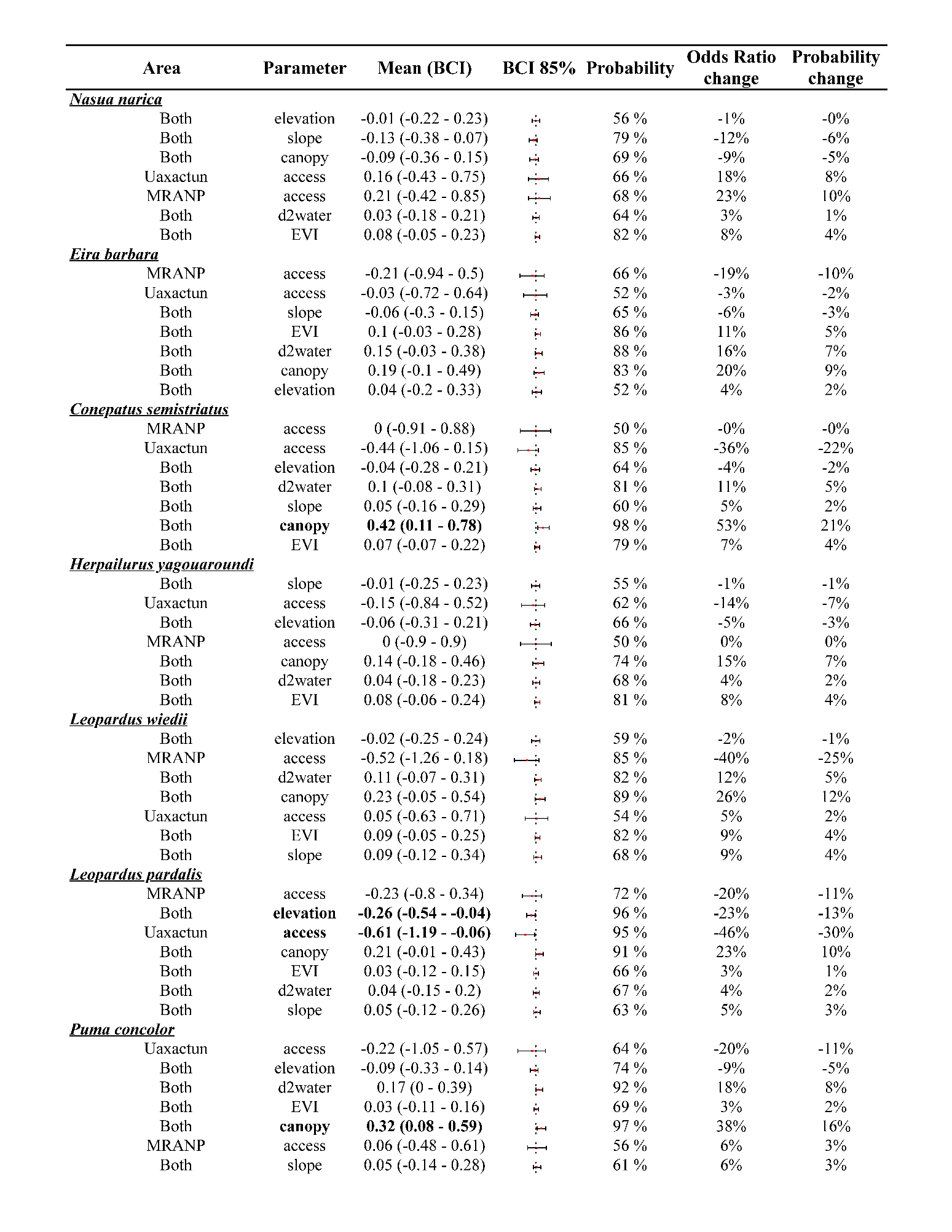


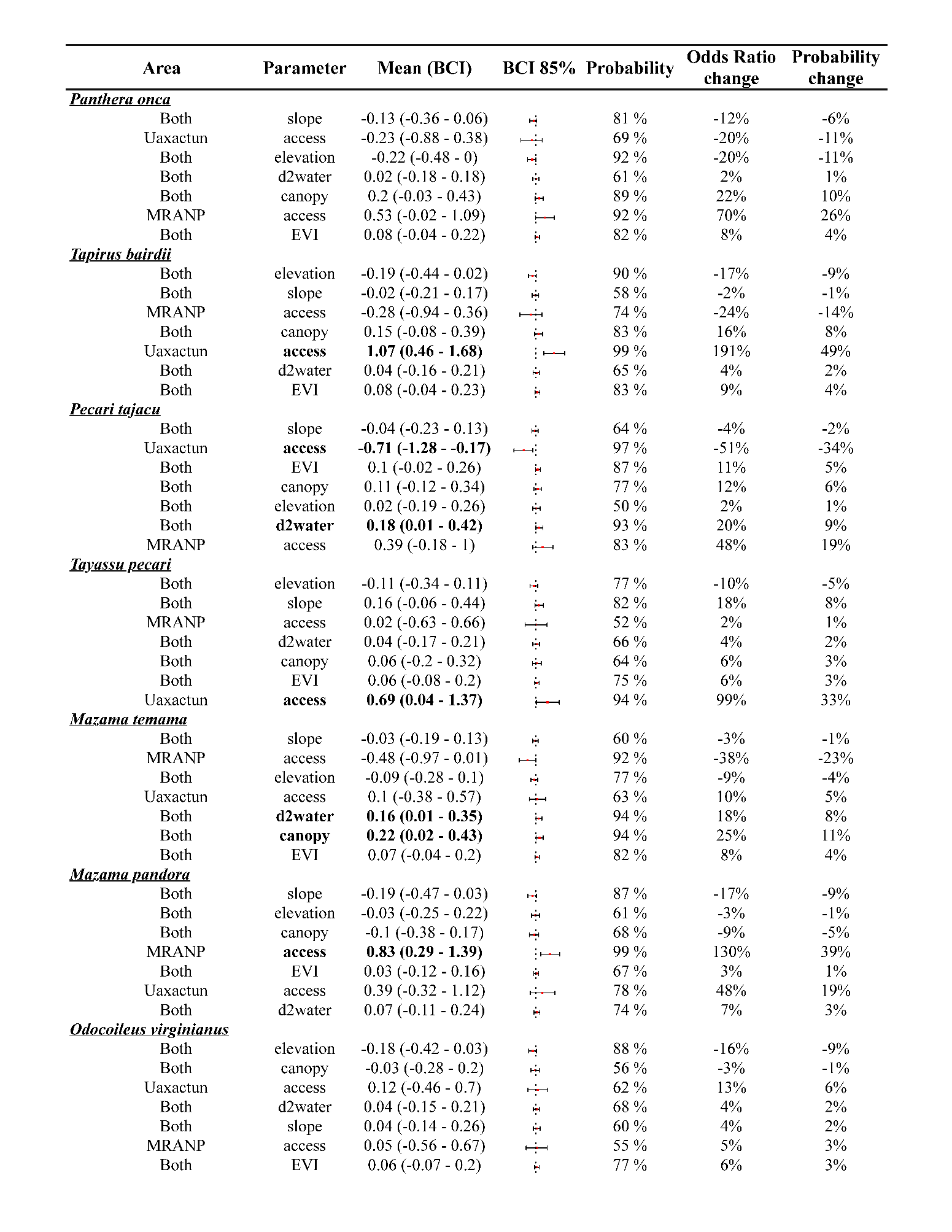


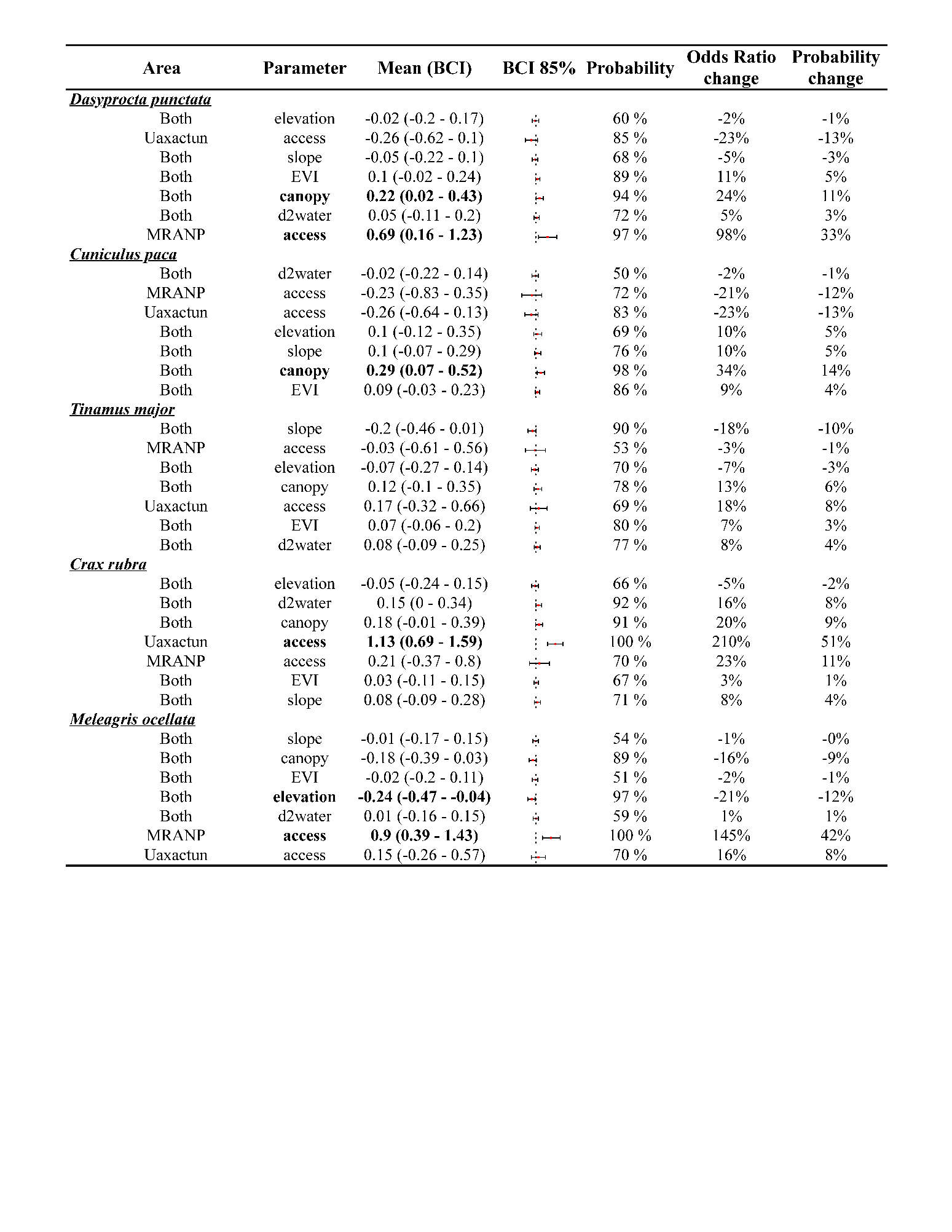


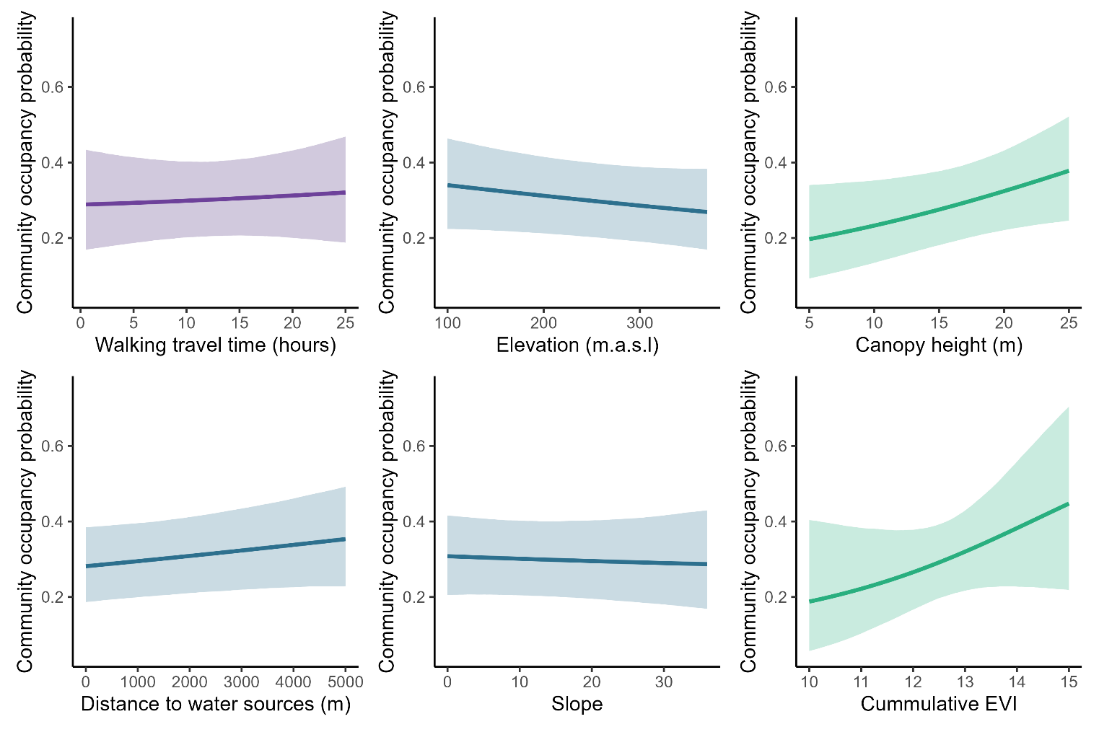


Appendix S8. Predictions for the effects of covariates on the mean occupancy probability for the entire surveyed community across Uaxactun and MRANP.

Appendix S9. Detection and occupancy hyperparameter estimate for the entire community surveyed across Uaxactun and Mirador Rio Azul National Park.


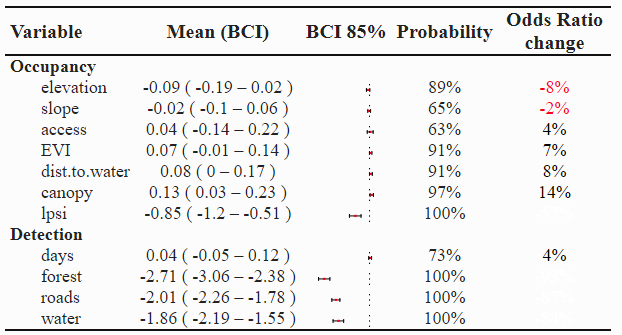


Appendix S10. Parameter estimates for elevation, slope, and distance to water effects on occupancy probability, with linewidth representing 66% and 85% Bayesian credible intervals.


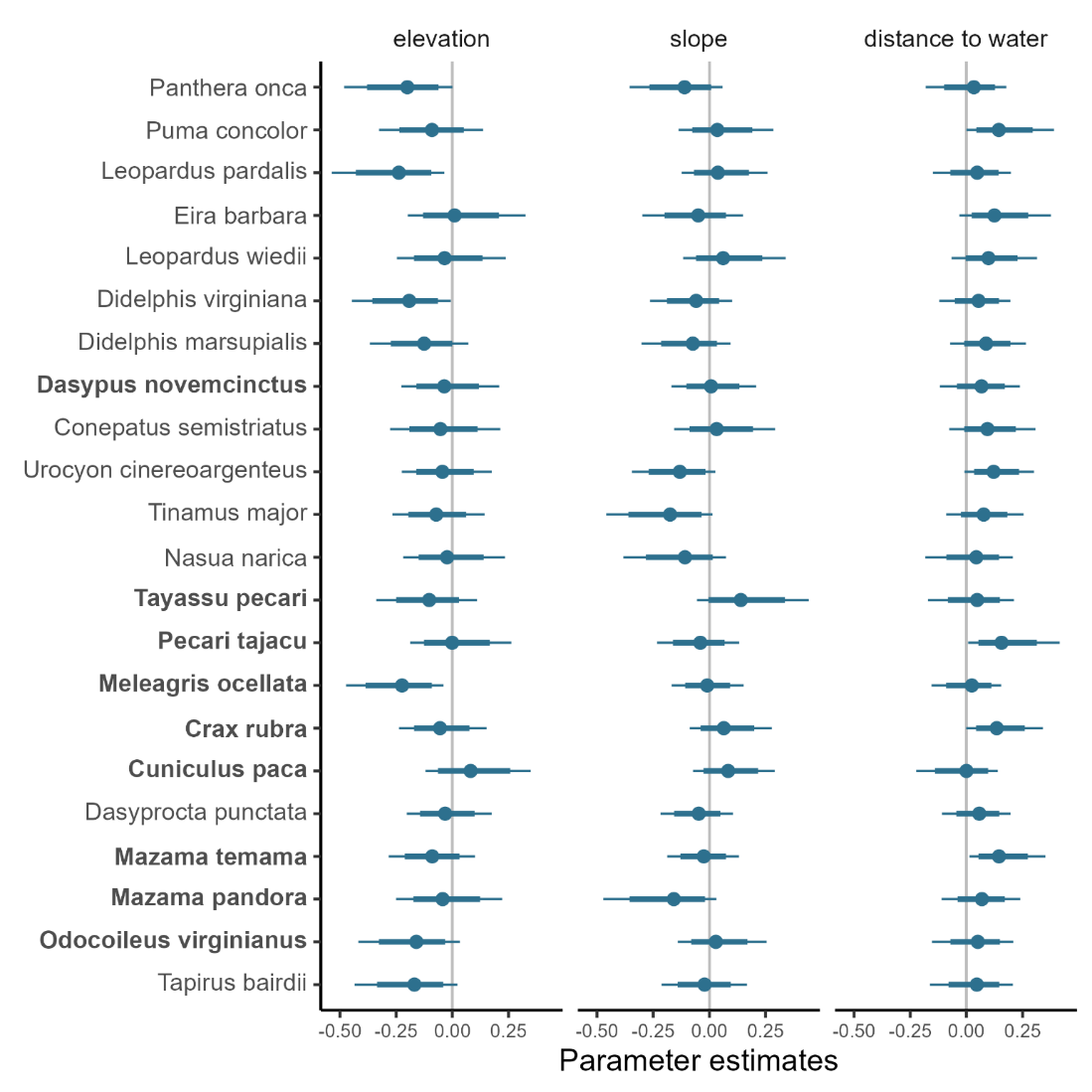


Appendix S11. Parameter estimates for canopy height (m) and the Enhanced Vegetation Index (EVI) on occupancy probability, with linewidth representing 66% and 85% Bayesian credible intervals.


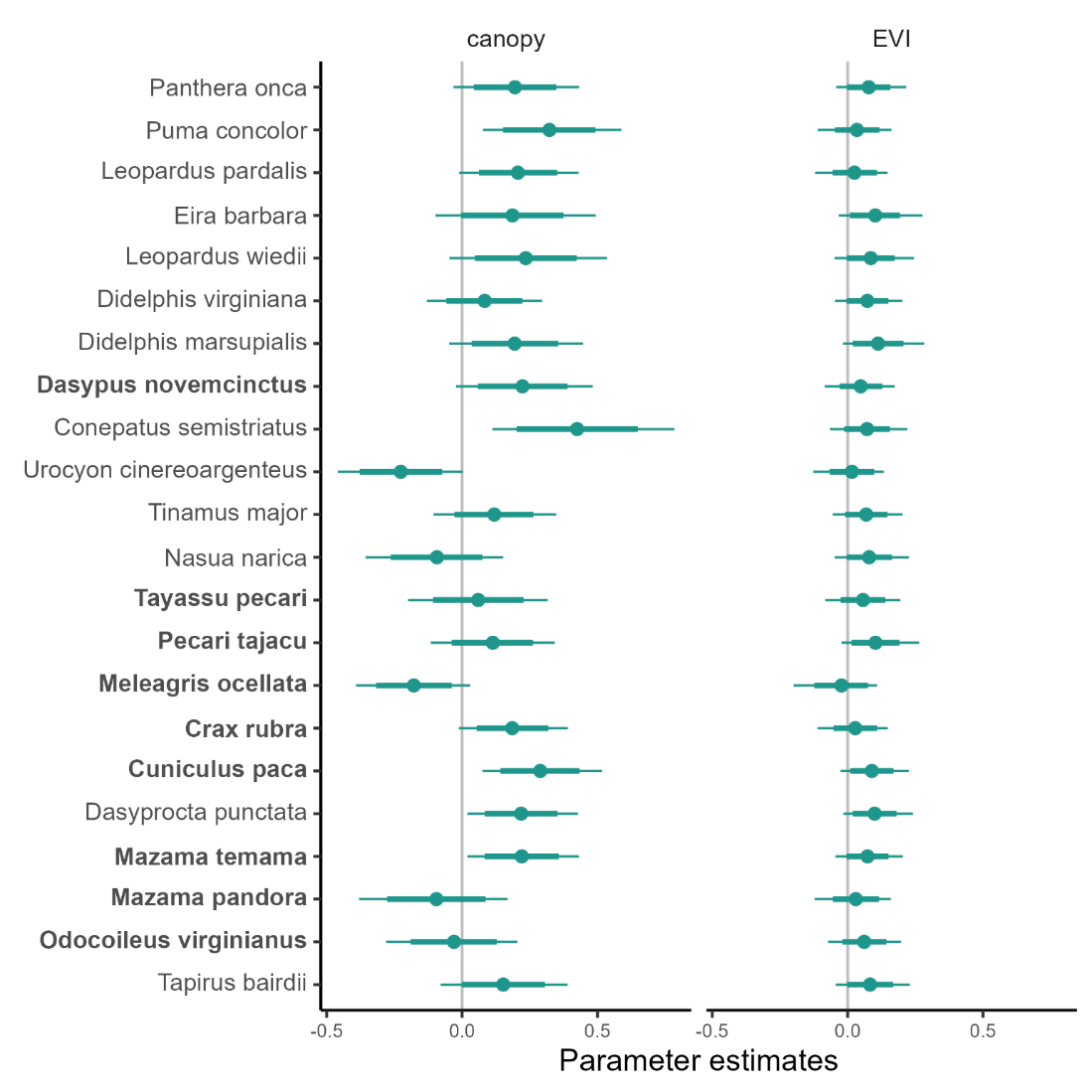


Appendix S12. Mean estimates (85% and 66% BCI) for the effect of seasonality on detection probability.


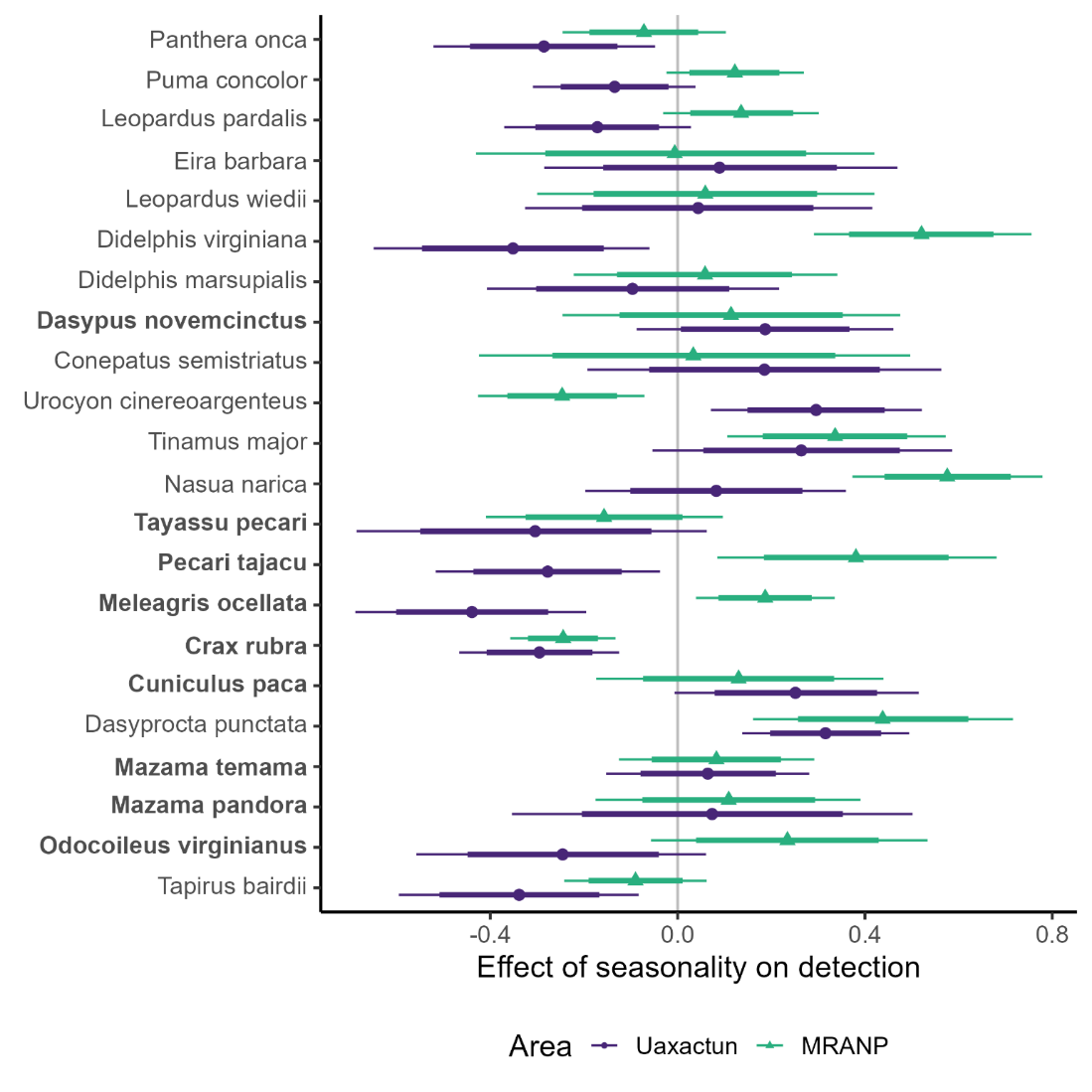


Appendix S13. Mean point estimates and their 85% Bayesian Credible Interval (BCI) for detection (intercept) on different camera trap locations, and the effect of seasonality on detection (slope). Bold parameters indicate significantly higher estimates in MRANP. Seasonality is measured as Julian days. Probability refers to the probability that the parameter is either positive or negative, referring to the percentage of the posterior distribution that does not overlap zero.


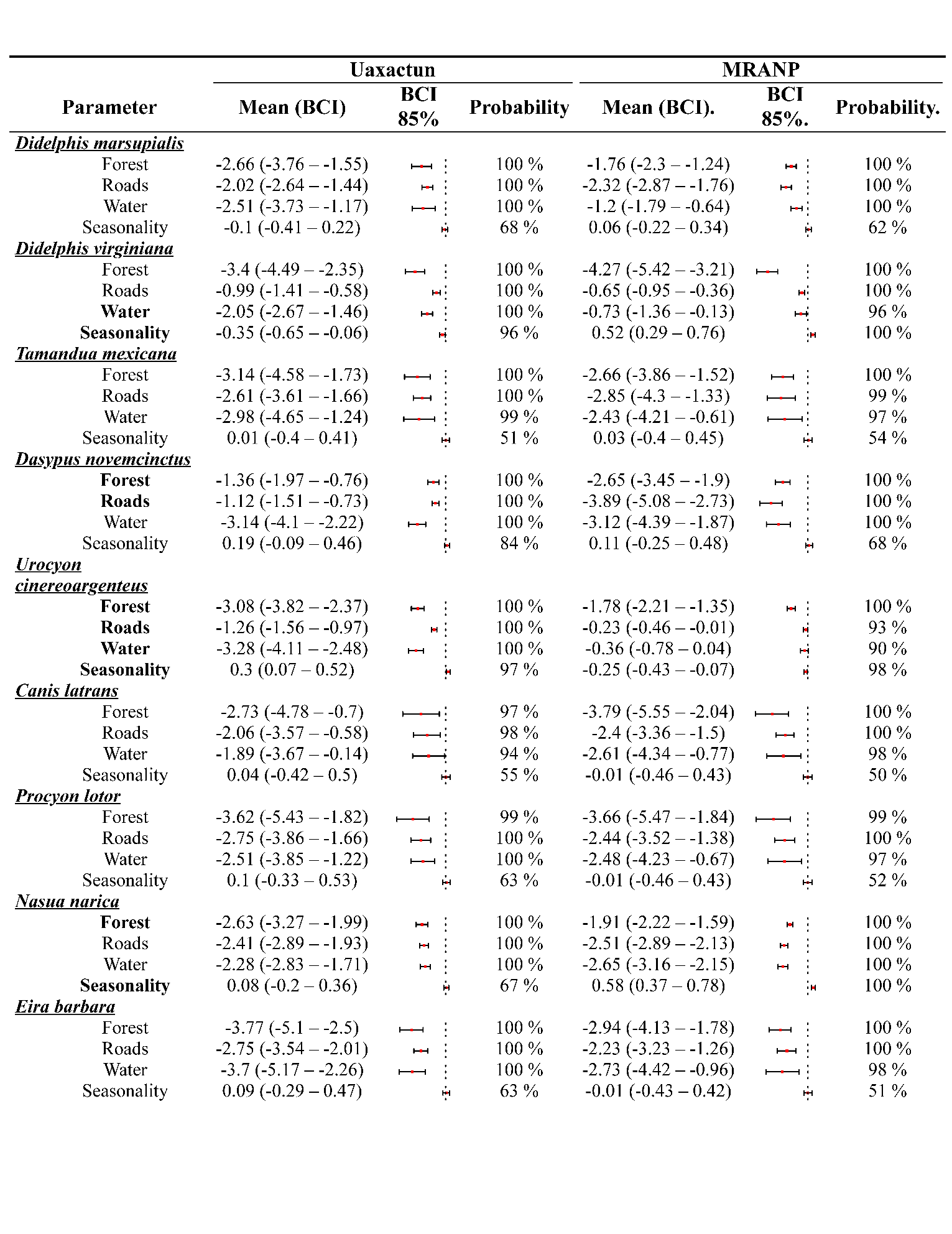


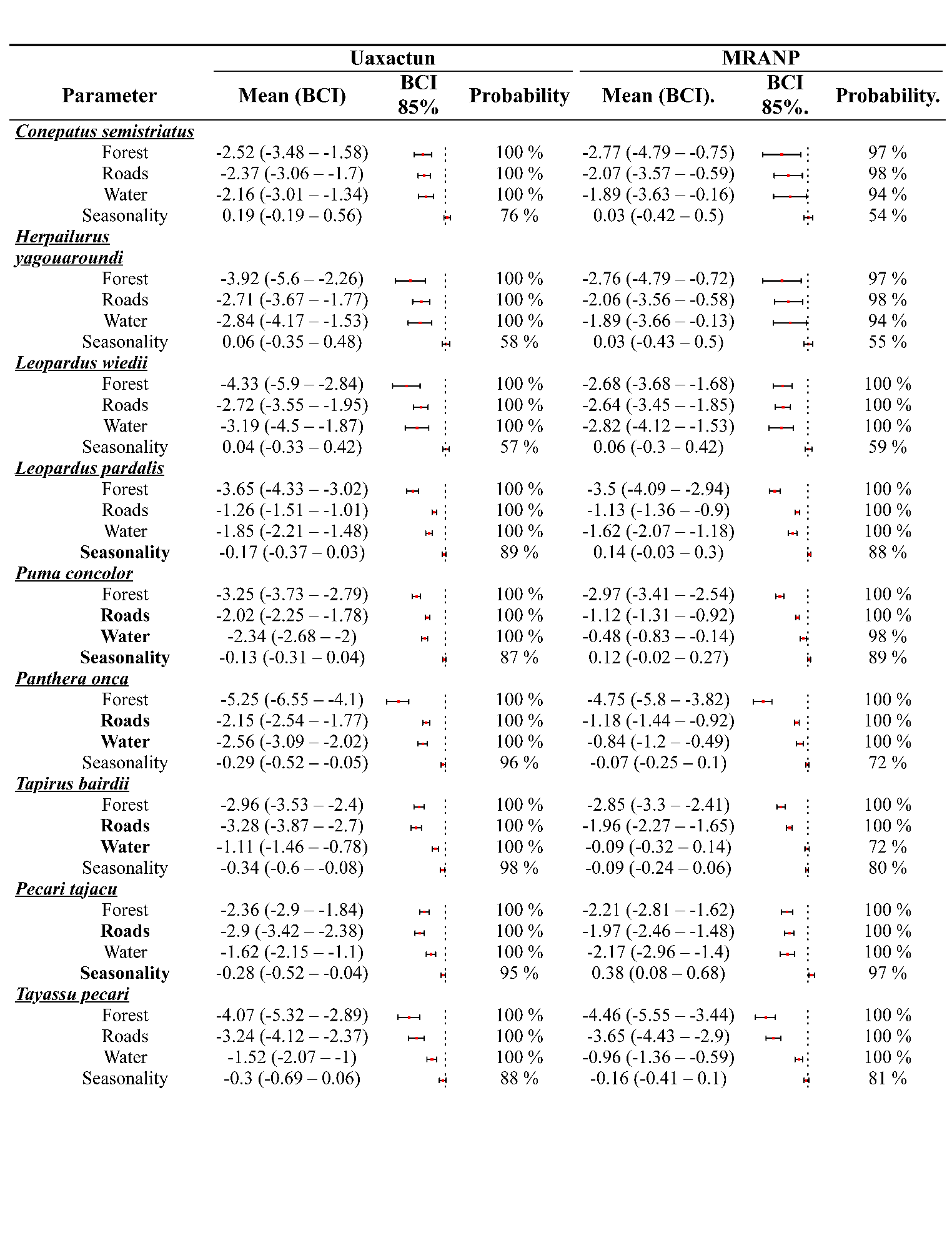


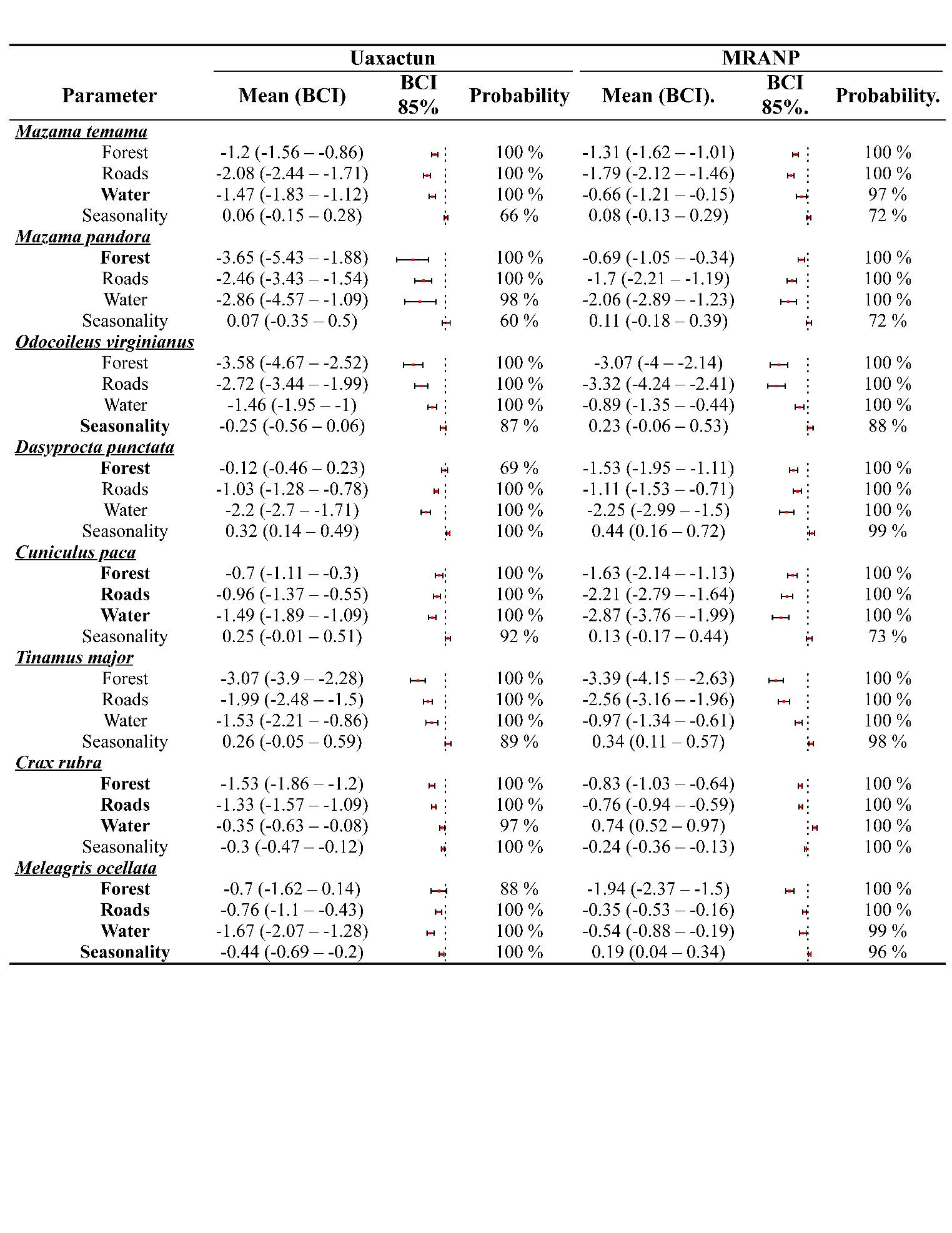


Appendix S14. Mean detection estimates (85% and 66% BCI) in the different types of locales where cameras were placed.


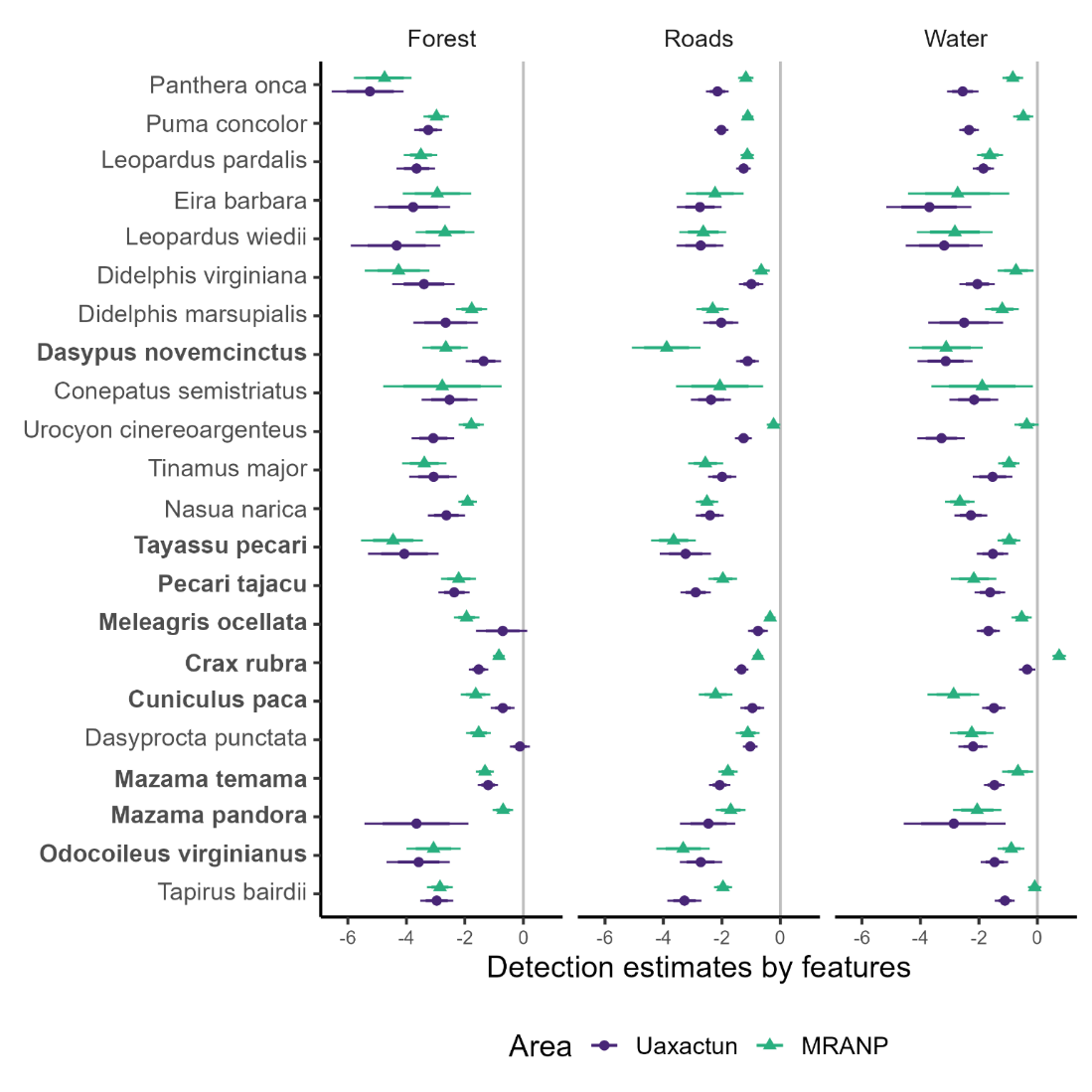


Appendix S15. Differences in detection probability within a pair of features. Colors indicate features with contrasting detection probability, whereas the line type indicates the area where the difference occurred.


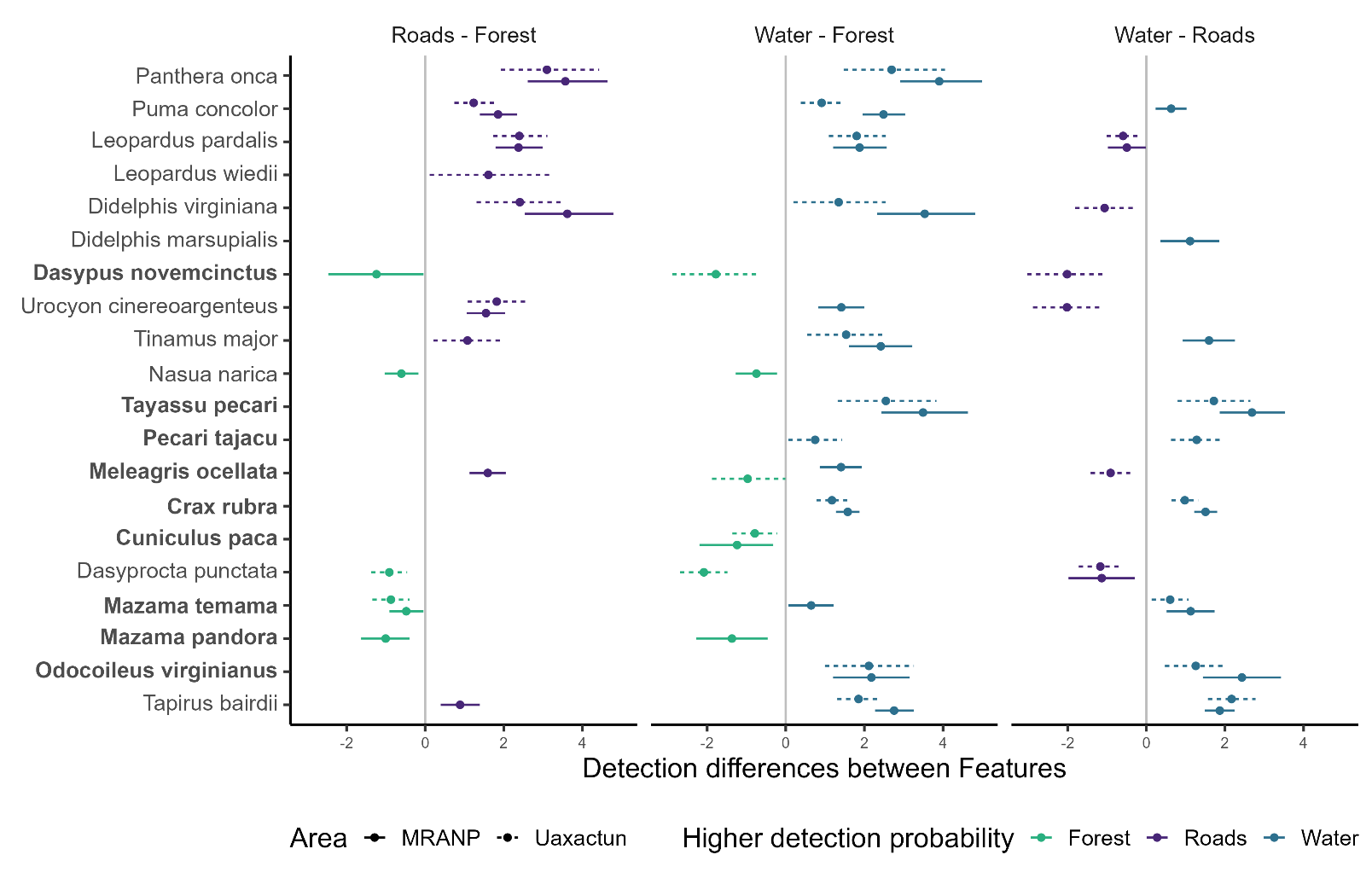


Appendix S16. Differences in detection probability between areas per feature and species. The higher detection probability on the right indicates a higher detection estimate in the protected area (light color) and in Uaxactun otherwise (dark color). The graph omit species with no differences in detection probabilities between the two management units.


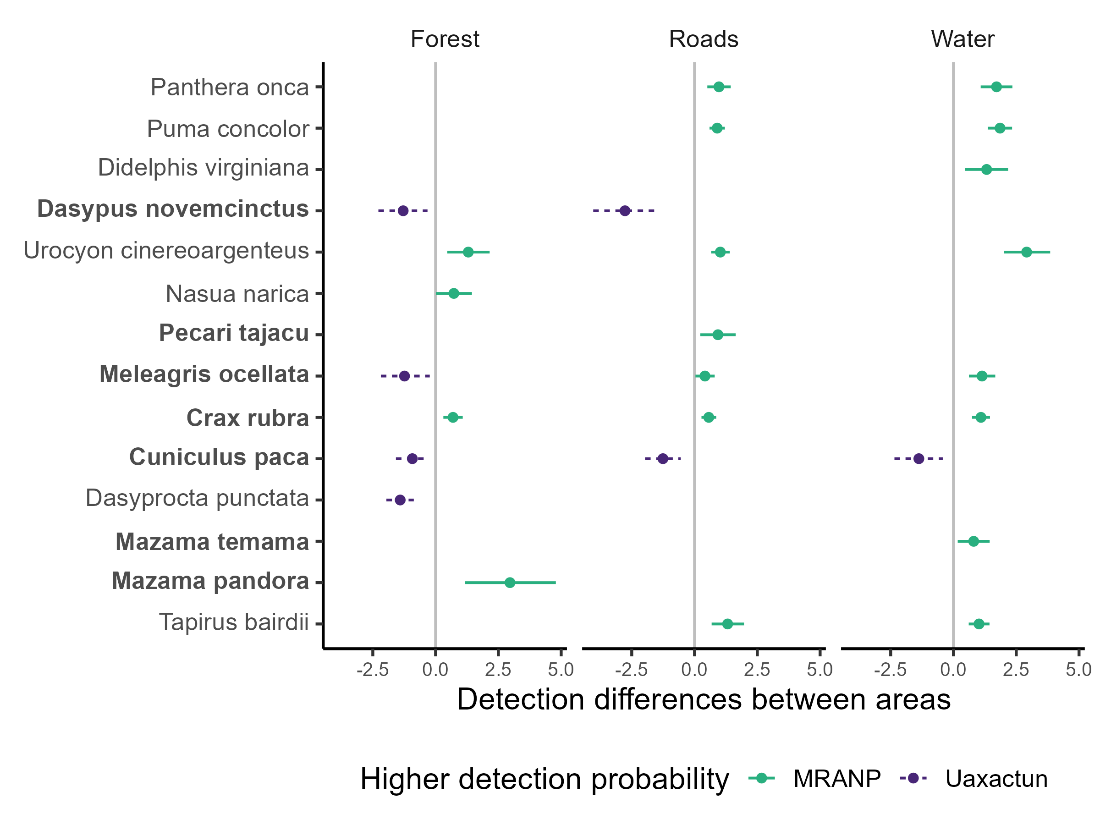


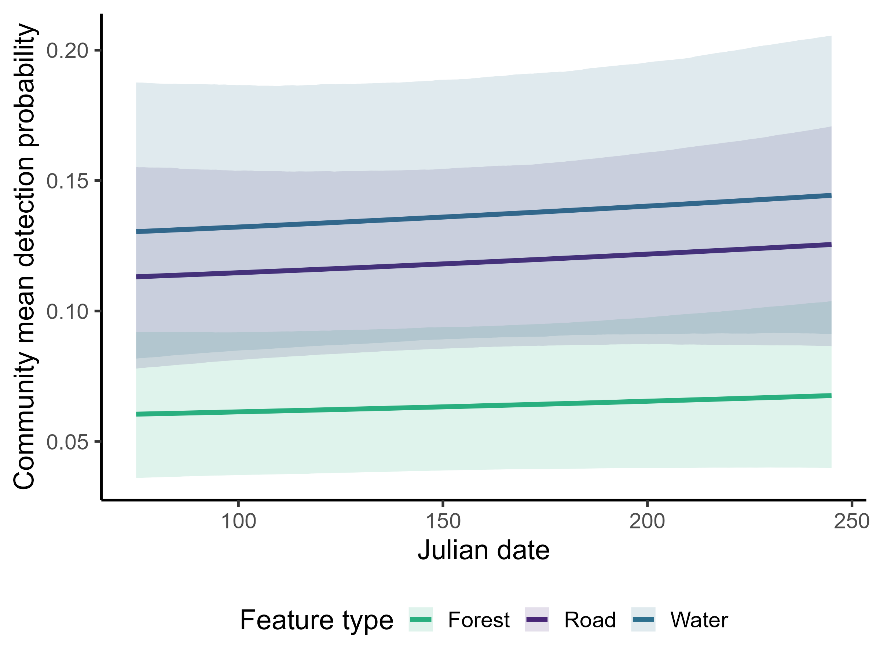
Appendix S17. Predictions for the mean community detection probability. Overall, cameras placed in water sources and roads had higher detection probabilities than those placed in the forest. The survey date had a weak but positive effect on the detection probability through the season, with a 68% percent probability of having higher detection at the end of the dry season. We can see that overall detection probability was low at the community level.


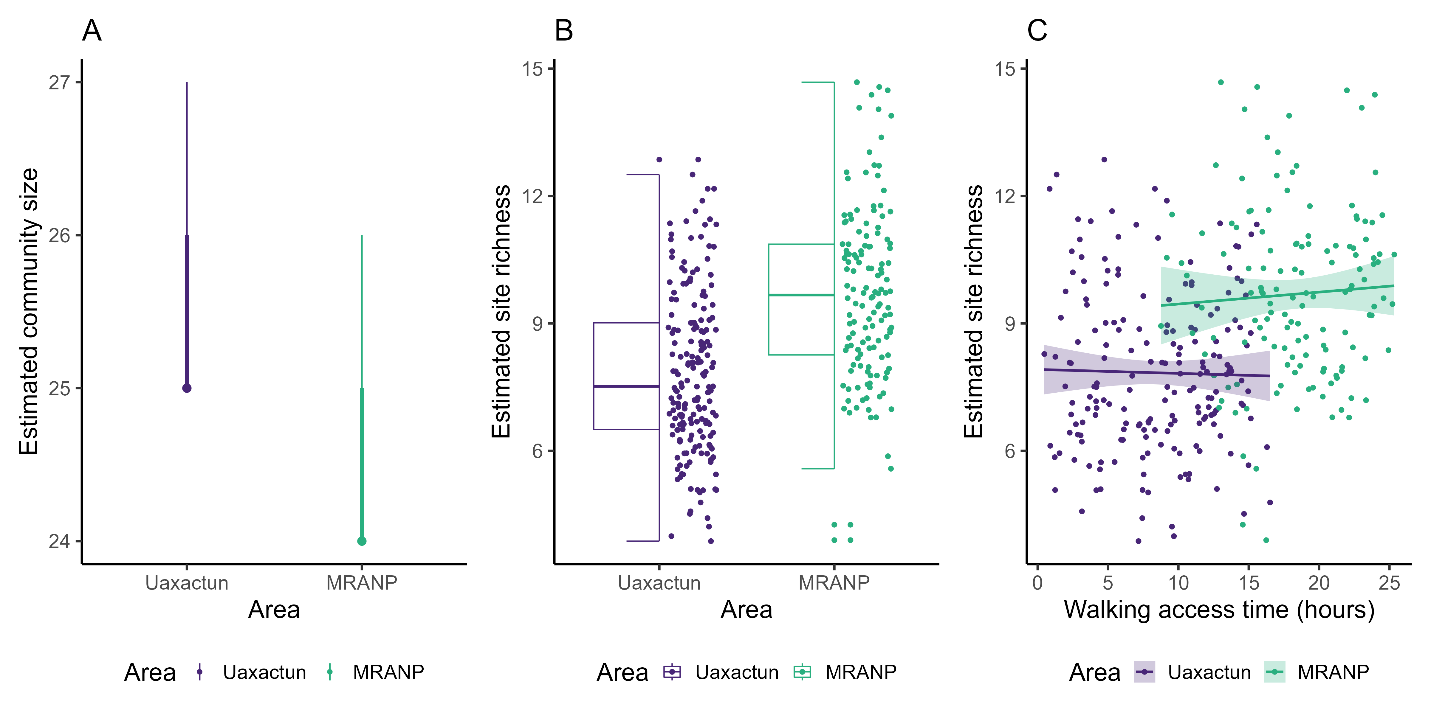


Appendix S18. Estimated richness per area (A) and sites (camera trap locations) within areas (B). Although the overall site richness mean is higher in MRANP, neither the estimated community size nor the richness per site differs from Uaxactun estimates (i.e., BCI overlaps in both cases). Although there was a great variation in site richness within both areas (C), there was no relationship with walking access time.

1. The probability of MRANP being higher than Uaxactun is represented by more intense colors. More intense green and purple colors indicate greater probabilities of occupancy in the protected areas and Uaxactun, respectively. In relative importance of access, more intense colors indicate greater importance of access in any given area. [↑](#footnote-ref-1)
